# Early and Sustained Asthma Control and Remission in Real‐World Patients With Severe Eosinophilic Asthma Treated With Benralizumab: XALOC‐2

**DOI:** 10.1111/cea.70162

**Published:** 2025-10-29

**Authors:** Erika Penz, Thomas Rothe, Lieven Dupont, Trung N. Tran, Andrew Menzies‐Gow, Anat Shavit, David Cohen, Tanja Plate, Sheena Kayaniyil, An Herreman, Claudio Schuoler, Benjamin Emmanuel, Marek Lommatzsch

**Affiliations:** ^1^ University of Saskatchewan Saskatoon Saskatchewan Canada; ^2^ Cantonal Hospital Graubünden Chur Switzerland; ^3^ University Hospital of Leuven Leuven Belgium; ^4^ AstraZeneca Gaithersburg Maryland USA; ^5^ AstraZeneca Cambridge UK; ^6^ AstraZeneca Wedel Germany; ^7^ AstraZeneca Mississauga Ontario Canada; ^8^ AstraZeneca Groot‐Bijgaarden Belgium; ^9^ AstraZeneca Baar Switzerland; ^10^ University of Rostock Rostock Germany

**Keywords:** asthma control, benralizumab, clinical remission, eosinophils, severe asthma

## Abstract

**Background:**

Prospective real‐world data concerning the early and sustained effects of benralizumab on asthma control in patients with severe eosinophilic asthma (SEA) is lacking.

**Methods:**

XALOC‐2 is a prospective, observational, multi‐national, real‐world study in adults with SEA treated with benralizumab. This integrated analysis assessed Asthma Control Questionnaire (ACQ) scores, achievement of 3‐component clinical remission (which included well‐controlled symptoms [ACQ score ≤ 0.75], no exacerbations, and no use of maintenance oral corticosteroids [mOCS]), and other clinical outcomes, over a 12‐month baseline period and up to Week 56. Associations between remission status and key baseline characteristics were also assessed.

**Results:**

535 patients were included. Median (interquartile range) ACQ score at baseline was 3.0 (2.2–3.8). At Week 1, 58.0% (282/486) of patients had ACQ score reductions of ≥ 0.5 points (minimal clinically important difference [MCID]) and 35.0% (170/486) had reductions of ≥ 1 point (2× MCID). By Week 56, these increased to 78.6% (276/351) and 62.1% (218/351), respectively. Improved asthma control after benralizumab initiation was similar irrespective of previous biologic use status. By Week 56, clinical remission criteria were achieved in 26.7% (70/262) of patients versus 0% (0/374) at baseline. No mOCS use, lower body mass index, better asthma symptom control and higher peak blood eosinophil count at baseline were associated with meeting 3‐component clinical remission criteria at Week 56.

**Conclusions:**

Real‐world patients receiving benralizumab showed early and sustained improvements in asthma symptoms, regardless of previous biologic use. More than a quarter of patients achieved clinical asthma remission after 1 year of benralizumab treatment.

## Graphical abstract

Real‐world patients with severe eosinophilic asthma treated with benralizumab showed early and sustained improvements in symptoms. Clinically meaningful improvements began as early as one week after benralizumab initiation, regardless of previous biologic use, and further improved over the following year. The 3‐component clinical remission criteria were met in more than a quarter of patients at Week 56.

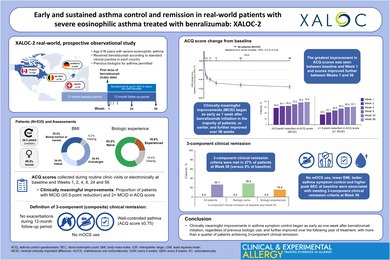

**Summary**

- Benralizumab‐treated real‐world patients with severe eosinophilic asthma showed early and sustained clinically meaningful improvements in symptoms.
- 3‐Component clinical remission was achieved in more than a quarter of patients at Week 56.
- Targeting eosinophilic inflammation is associated with meaningful responses across patients with key clinically relevant characteristics.

## 1 Introduction

At least 80% of patients with severe asthma are likely to have an eosinophilic phenotype [1]. Severe eosinophilic asthma (SEA) is characterised by an interleukin‐5 subunit (IL‐5)‐mediated, type‐2 inflammatory cascade, which is associated with blood and tissue eosinophilia [1, 2]. Recently developed eosinophil‐targeting biologics have shown promise in decreasing blood and tissue eosinophil counts [3, 4, 5], with associated improvements in measures of SEA severity [5, 6, 7]. Benralizumab (a humanised, afucosylated, monoclonal antibody) has demonstrated the capacity to produce rapid, near‐complete depletion of eosinophils in blood [8], with subsequent near‐complete depletion in tissue. Benralizumab targets the IL‐5 receptor α subunit with high affinity and specificity and, additionally, induces eosinophil apoptosis through antibody‐dependent, cell‐mediated cytotoxicity [3], leading to a substantial improvement in asthma symptoms [6, 7, 9, 10, 11].

Guidelines now recommend the use of biologics, including benralizumab, for the treatment of SEA [12, 13, 14]. The approval of biologics has transformed the treatment landscape for SEA [15], making clinical remission a viable and meaningful treatment goal [16, 17]. An expert consensus framework published in 2020 proposed four key criteria to define asthma remission over a 12‐month period: (i) sustained absence of significant symptoms by a validated instrument; (ii) optimisation and stabilisation of lung function; (iii) patient/provider agreement regarding disease remission; and (iv) no use of systemic corticosteroid therapy for exacerbation treatment or long‐term disease control [16]. However, definitions of clinical remission vary across the literature. While the most commonly used definition includes three components: asthma control, no exacerbations, and no systemic corticosteroid use for a period of at least 12 months, some frameworks incorporate a fourth component of stable or normal lung function [18]. A recent systematic review and meta‐analysis identified 48 definitions of clinical remission across 25 studies, underscoring the lack of consensus in the field [17].

The Severe Asthma Network Italy Delphi consensus defined complete remission as the absence of symptoms and exacerbations, no use of maintenance oral corticosteroids (mOCS), and stable lung function [19]. The International Severe Asthma Registry proposed a multidomain approach to remission, incorporating exacerbation rate, long‐term OCS use, asthma control, and lung function [20]. Additionally, a multidisciplinary workgroup of asthma experts published a consensus statement advocating for a more stringent six‐component definition, including no symptoms requiring one‐time reliever therapy more than once a month [21]. Despite this variability, most proposed definitions converge on three core criteria: no exacerbations, no OCS use, and asthma symptom control [18]. In this study, we adopted the 3‐component definition, aligning with guideline‐endorsed criteria [21] and previous real‐world studies, while recognising the ongoing debate regarding the most appropriate endpoints for defining remission in severe asthma. Although different thresholds and tools are currently used in the literature to define asthma control in the context of remission (Asthma Control Test [ACT] score ≥ 20 or ≥ 23; Asthma Control Questionnaire [ACQ] score < 1.5 or ≤ 0.75) [17], we decided to use one of the strictest criteria for asthma control (well‐controlled asthma; ACQ score ≤ 0.75) [18].

While the efficacy and safety of benralizumab have been established by several randomised controlled trials (RCTs), such as SIROCCO [9], CALIMA [10], and ZONDA [11], real‐world data are needed to prospectively elucidate the effectiveness of benralizumab treatment in patients with severe asthma. Results of the retrospective XALOC‐1 program in real‐world patients with SEA treated with benralizumab showed clinically meaningful improvements in ACQ scores, which exceeded the minimal clinically important difference (MCID [≥ 0.5 point reduction]), as well as substantial improvements in clinical outcomes, irrespective of previous biologic use and key clinical characteristics [7, 22]. In addition, lower body mass index (BMI) was positively associated with clinical remission, in line with previous studies that reported that patients with higher BMI are more likely to have comorbidities and often report worse ACT and ACQ scores, regardless of asthma status [23, 24].

Here, we report the results of XALOC‐2, the first prospective, multinational real‐world study of benralizumab in SEA.

## 2 Methods

### 2.1 Study Design

XALOC‐2 is a prospective, observational real‐world study composed of four national studies (POWER [Canada], BE‐REAL [Belgium], imPROve [Germany], and BEEPS [Switzerland]) that investigated adults with SEA who received benralizumab for up to 56 weeks (November 2018 to December 2023).

Benralizumab 30 mg was administered subcutaneously (SC) every 4 weeks for the first three doses and every 8 weeks thereafter in line with the approved dosage and standard clinical practice. Initiation of benralizumab was decided independently by the investigator and was not driven by the study protocol. Study design, inclusion criteria, and data collection were standardised across the national studies, allowing integration of patient‐level data.

Baseline was defined as the 12‐month period before the index date (Week 0, first administration of benralizumab). The follow‐up period for the current analysis was up to 56 weeks from the index date (Figure S1). The patients' clinical and demographic data were extracted from relevant paper and/or electronic medical records, where available.

Studies were performed in accordance with the Declaration of Helsinki, the International Conference on Harmonisation Good Clinical Practice guidelines, Good Pharmacoepidemiology Practice, and all applicable legislation on non‐interventional studies and/or observational studies. Ethical approval for the studies was provided by the appropriate institutional review board and/or independent ethics committee for each clinical site. Informed consent practice varied across the individual studies, per local legislation.

### 2.2 Study Participants

Adults (≥ 18 years) with SEA who initiated benralizumab were included in this integrated analysis. Inclusion criteria, as defined in each national study, are described in Supporting Information: Methods S1.

Patients were categorised based on the following key baseline clinical characteristics, as available: previous biologic use, exacerbation history, peak blood eosinophil count (BEC), mOCS use, age at asthma diagnosis, presence of chronic rhinosinusitis with nasal polyposis (CRSwNP), atopic status, fractional exhaled nitric oxide (FeNO), and BMI (Supporting Information: Methods S2).

### 2.3 Outcome Measures

ACQ score (0 [totally controlled] to 6 [severely uncontrolled]) was collected either during routine clinic visits or directly from patients (on paper, telephone or electronically) for Week 0 and Weeks 1, 2, 4, 8, 16 or 24, and 56, using either the five‐ or six‐item version of the questionnaire (ACQ‐6 in POWER [Canada], BE‐REAL [Belgium], and imPROve [Germany], and ACQ‐5 in BEEPS [Switzerland]; Supporting Information: Methods S2).

Clinical outcomes measured at baseline and at Weeks 24 and 56 were the number of asthma exacerbations, mOCS use (and daily dose), and, when part of routine clinical practice in the hospital or clinic, pre‐/post‐bronchodilator forced expiratory volume in 1 s (FEV_1_). Definitions of asthma control (including MCID), exacerbations, annualised exacerbation rate (AER), mOCS use and changes in dose, lung function outcomes, and peak BEC are provided in Supporting Information: Methods S2.

Clinical remission was defined as the composite of three individual components: asthma symptom control (ACQ score ≤ 0.75 [and < 1.5 as a sensitivity analysis]), no exacerbations, and no OCS use [25]. Exacerbations were assessed during the 12 months prior to index (baseline period) and from the index date to Week 56 (follow‐up period). Asthma symptom control and mOCS use status were assessed at index (Week 0) and Week 56. Lung function was not included in the remission criteria owing to limited availability of data.

Additionally, in BE‐REAL and imPROve, nasal polyposis symptoms were evaluated using an exploratory visual analog scale (VAS) of 1–10 (1, not bothersome at all; 10, extremely bothersome, over the previous month) and are reported for index and for Weeks 24 and 56 among patients with comorbid nasal polyps at baseline.

Benralizumab treatment patterns (duration, discontinuation, and reasons for discontinuation) were captured over the 56‐week follow‐up period.

Lung function data were available at index in BE‐REAL (Belgium) and imPROve (Germany) collected using spirometry, and in BEEPS (Switzerland) collected using spirometry and daily peak expiratory flow measurements. Lung function data were available at Weeks 24 and 56 in BE‐REAL and BEEPS, when part of routine clinical practice.

### 2.4 Statistical Analysis

Descriptive statistics, including mean, median, standard deviation (SD), and range for continuous variables, and number and percentage of patients for categorical variables, are presented for baseline demographics and clinical characteristics, overall and by previous biologic use; only non‐missing values are presented.

Longitudinal mixed models with repeated measures were used to summarise continuous endpoints over time with estimated least square means (LSM) and corresponding 95% confidence intervals (CIs) for change from baseline in ACQ score. The percentage of patients achieving MCID (≥ 0.5‐point reduction) in ACQ score was calculated, and CIs were determined using binomial Clopper–Pearson exact 95% CIs.

The proportion of patients meeting the 3‐component clinical remission definition (among those who had all three components available) at each time point, and each individual component of remission, is presented as number and percentage at baseline and Week 56, overall, by patients' previous biologic use and by key clinical demographics and characteristics at baseline (exacerbation history, age at asthma diagnosis, FeNO, BEC, mOCS use, atopic status, CRSwNP status, and BMI).

We used univariable and multivariable logistic regression analyses to assess the association between key baseline demographics and clinical characteristics, and clinical remission status (using the less stringent criterion of ACQ score < 1.5), at Week 56. Statistical significance was determined using log‐likelihood ratios. Core baseline variables (sex, age at start of benralizumab treatment [continuous, based on 5‐year increments], exacerbations during baseline [continuous, based on one‐unit increments], mOCS dose [mg/day, based on 5 mg/day increments], controlled asthma [ACQ score < 1.5], and asthma duration [continuous, based on 10‐year increments]) were inputted into a multivariable regression model. Additional baseline variables found to be significant by univariable analysis (*p* < 0.05) were selected for inclusion in the multivariable model using a backward selection method based on a change of 10% or more in the 95% CI: (1) for at least one core independent variable or (2) for ≥ 80% of all independent variables. Results are presented as odds ratios (ORs) with 95% CIs and *p*‐values. Comparisons were two‐sided, and significance was considered at an *α* level of 0.05.

## 3 Results

### 3.1 Patients and Benralizumab Treatment

A total of 535 patients were eligible for inclusion in the integrated analysis (Table 1, imPROve [Germany], *n* = 244 [45.6%]; POWER [Canada], *n* = 142 [26.5%]; BE‐REAL [Belgium], *n* = 76 [14.2%]; and BEEPS [Switzerland], *n* = 73 [13.6%]).

**TABLE 1:** Baseline demographics and disease characteristics, for the overall population and according to patients' previous biologic experience.^a^

Baseline demographics and disease characteristics	All patients (*N* = 535)	Biologic‐naïve patients (*n* = 348)^a^	Biologic‐experienced patients (*n* = 89)^a^
Age at initiation of benralizumab, years			
Median (IQR)	58.0 (48.0–66.0)	58.0 (49.0–66.0)	55.0 (47.0–64.0)
Age group, *n* (%)			
< 65 years	372 (69.5)	248 (71.3)	67 (75.3)
≥ 65 years	152 (28.4)	94 (27.0)	22 (24.7)
Missing	11 (2.1)	6 (1.7)	0 (0)
Age at asthma diagnosis, years^b^	(*n* = 376)	(*n* = 296)	(*n* = 80)
Median (IQR)	38.0 (21.0–53.0)	40.5 (21.0–55.0)	31.5 (19.5–45.5)
Age at asthma diagnosis groups, *n* (%)^b^	(*n* = 393)	(*n* = 310)	(*n* = 83)
< 18 years	76 (19.3)	58 (18.7)	18 (21.7)
≥ 18 years	300 (76.3)	238 (76.8)	62 (74.7)
≥ 18 to < 40 years	119 (30.3)	89 (28.7)	30 (36.1)
≥ 40 years	181 (46.1)	149 (48.1)	32 (38.6)
Sex, female, *n* (%)	263 (49.2)	181 (52.0)	52 (58.4)
Missing	11 (2.1)	6 (1.7)	0 (0)
Participating country, *n* (%)			
Germany (imPROve)	244 (45.6)	201 (57.8)	43 (48.3)
Canada (POWER)	142 (26.5)	38 (10.9)	6 (6.7)
Belgium (BE‐REAL)	76 (14.2)	56 (16.1)	20 (22.5)
Switzerland (BEEPS)	73 (13.6)	53 (15.2)	20 (22.5)
BMI, kg/m^2^	(*n* = 502)	(*n* = 342)	(*n* = 88)
Median (IQR)	28.4 (24.7–32.3)	28.0 (24.5–31.6)	28.7 (25.2–32.1)
BMI group, kg/m^2^, *n* (% of *N*)	(*n* = 502)	(*n* = 342)	(*n* = 88)
Normal (< 25)^c^	134 (25.0)	101 (29.0)	21 (23.6)
Overweight (≥ 25 to < 30)	184 (34.4)	129 (37.1)	36 (40.4)
Obese (≥ 30)	184 (34.4)	112 (32.2)	31 (34.8)
Missing	33 (6.2)	6 (1.7)	1 (1.1)
Smoking history, *n* (%)			
Never smoker	300 (56.1)	201 (57.8)	60 (67.4)
Current smoker	30 (5.6)	23 (6.6)	3 (3.4)
Previous smoker	170 (31.8)	117 (33.6)	25 (28.1)
Missing	35 (6.5)	7 (2.0)	1 (1.1)
Comorbidities, *n* (% of *n* with available)			
Allergy	134 (25.0)	66 (19.0)	11 (12.4)
Cardiovascular diseases	90 (16.8)	42 (12.1)	17 (19.1)
Cataracts	34 (6.4)	8 (2.3)	3 (3.4)
Chronic rhinosinusitis^b^	112 (28.5)	83 (26.8)	29 (34.9)
Chronic rhinosinusitis with nasal polyposis^d^	157 (29.3)	104 (29.9)	28 (31.5)
Depression/anxiety	57 (10.7)	36 (10.3)	10 (11.2)
Eczema	34 (6.4)	21 (6.0)	1 (1.1)
Gastro‐oesophageal reflux disease	77 (14.4)	45 (12.9)	17 (19.1)
Glaucoma^e^	9 (2.3)	5 (2.1)	0 (0)
Obstructive sleep apnea	45 (8.4)	30 (8.6)	13 (14.6)
Metabolic diseases^b^	23 (5.9)	16 (5.2)	7 (8.4)
Osteoporosis, osteopenia or history of fractures^b^	32 (8.1)	22 (7.1)	10 (12.0)
Type 2 diabetes^f^	36 (11.3)	25 (9.7)	11 (17.5)
Positive atopic status, *n* (%)^b^, ^g^	(*n* = 393), 72 (18.3)	(*n* = 310), 58 (18.7)	(*n* = 83), 14 (16.9)
mOCS use at index date, *n* (%)^h^	190 (35.5)	129 (37.1)	46 (51.7)
Daily mOCS dosage, mg/day^h^	(*n* = 189)^i^	(*n* = 128)^i^	(*n* = 46)
Median (IQR)	20.0 (10.0–40.0)	19.2 (7.5–35.2)	12.8 (10.0–27.5)
mOCS use during 12‐month baseline period before index date, *n* (%)	315 (58.9)	227 (65.2)	66 (74.5)
Pre‐BD FEV_1_, L; median (IQR)^b^	(*n* = 385); 1.9 (1.4–2.5)	(*n* = 306); 1.9 (1.5–2.5)	(*n* = 79); 1.7 (1.2–2.4)
Post‐BD FEV_1_, L; median (IQR)^j^	(*n* = 56); 1.9 (1.5–2.5)	(*n* = 37); 2.1 (1.5–2.6)	(*n* = 19); 1.6 (1.2–2.1)
Predicted pre‐BD FEV_1_; *n* (%)^j^			
< 65%	(*n* = 76); 36 (47.4)	(*n* = 56); 28 (50.0)	(*n* = 20); 8 (40.0)
≥ 65%	(*n* = 76); 40 (52.6)	(*n* = 56); 28 (50.0)	(*n* = 20); 12 (60.0)
Exacerbations during 12‐month baseline period, median (IQR)^b^	(*n* = 389), 2.0 (1.0–4.0)	(*n* = 307), 2.0 (1.0–4.0)	(*n* = 82), 2.0 (1.0–4.0)
Exacerbations during 12‐month baseline period, *n* (%)^b^			
0–1	106 (27.0)	81 (26.1)	25 (30.1)
0	57 (14.5)	43 (13.9)	14 (16.9)
1	49 (12.5)	38 (12.3)	11 (13.3)
2	110 (28.0)	88 (28.4)	22 (26.5)
3	64 (16.3)	51 (16.5)	13 (15.7)
≥ 2	283 (72.0)	226 (72.9)	57 (68.7)
≥ 3	173 (44.0)	138 (44.5)	35 (42.2)
AER during 12‐month baseline period, median (IQR)^b^	3.0 (2.7–3.3)	3.0 (2.7–3.2)	3.3 (2.6–4.1)
Peak BEC during 12‐month baseline period, cells/μL; median (IQR)^b^	(*n* = 355); 530.0 (350.0–800.0)	(*n* = 278); 600.0 (400.0–860.0)	(*n* = 77); 400.0 (210.0–600.0)
Peak BEC group, cells/μL, *n* (%)^b^			
< 300	45 (11.5)	24 (7.7)	21 (25.3)
≥ 300	310 (78.9)	254 (81.9)	56 (67.5)
≥ 300 to < 500	112 (28.5)	85 (27.4)	27 (32.5)
≥ 500	198 (50.4)	169 (54.5)	29 (34.9)
≥ 1000	62 (15.8)	52 (16.8)	10 (12.1)
≥ 1500	18 (4.6)	17 (5.5)	1 (1.2)
FeNO, ppb (median, IQR)^b^	(*n* = 286); 33.5 (19.0–64.0)	(*n* = 221); 37.0 (19.0–66.0)	(*n* = 65); 26.0 (17.0–57.0)
FeNO ≥ 50 ppb; *n* (%)^b^	(*n* = 393); 91 (23.2)	(*n* = 310); 73 (23.5)	(*n* = 83); 18 (21.7)
FeNO ≥ 50 (ppb) and peak EOS ≥ 300 (cells/μL); *n* (%)^b^	77 (19.6)	64 (20.7)	13 (15.7)
Total serum IgE, IU/mL; median (IQR)^b^	(*n* = 323); 133.0 (44.7, 463.0)	(*n* = 260); 114.5 (41.9, 451.5)	(*n* = 63); 150.0 (52.0–472.0)
ACQ score at index (Week 0)^k^	(*n* = 516)	(*n* = 332)	(*n* = 86)
Mean (SD)	3.0 (1.2)	2.9 (1.1)	3.2 (1.5)
Median (IQR)	3.0 (2.2–3.8)	3.0 (2.2–3.7)	3.3 (1.8–4.3)

Abbreviations: ACQ, Asthma Control Questionnaire; ACQ‐5, five‐item Asthma Control Questionnaire; ACQ‐6, six‐item Asthma Control Questionnaire; AER, annualised exacerbation rate; BD, bronchodilator; BEC, blood eosinophil count; BMI, body mass index; CRSwNP, chronic rhinosinusitis with nasal polyposis; FeNO, fractional exhaled nitric oxide; FEV_1_, forced expiratory volume in one second; IgE, immunoglobulin E; IL‐4/13R, interleukin‐4 subunit/IL‐13 receptors; IL‐5, interleukin‐5 subunit; IQR, interquartile range; mOCS, maintenance oral corticosteroids; ppb, parts per billion; SD, standard deviation.

^a^ Previous biologic therapy (any previous use) included mepolizumab (*n* = 40/63), omalizumab (*n* = 18/63), dupilumab (*n* = 8/63), and reslizumab (*n* = 4/63; available in BEEPS [Switzerland] and imPROve [Germany] only, *n* = 63); *n* = 20 had received either an anti‐IL‐5, anti‐IgE, or anti‐IL‐4/IL‐13R (available in BE‐REAL [Belgium] only, *n* = 20); there were 98 patients from POWER (Canada) with biologic status missing.

^b^ Available in BE‐REAL (Belgium), BEEPS (Switzerland) and imPROve (Germany) only.

^c^ Includes nine patients with BMI < 18.5 kg/m^2^.

^d^ Nasal polyposis was evaluated at Week 24 and 56 in patients with nasal polyposis or CRSwNP at baseline only.

^e^ Available in POWER (Canada) and imPROve (Germany) only.

^f^ Available in BE‐REAL (Belgium) and imPROve (Germany) only.

^g^ Confirmed by skin prick test, clinical presentation or anamnesis in BEEPS (Switzerland), by history of positive allergy test (Y/N), and total IgE based on data available in medical records in BE‐REAL (Belgium), and by presence of atopic dermatitis or positive atopic status based on data available in medical records in imPROve (Germany).

^h^ mOCS dose was calculated as the patient's mean daily dosage over the past 30 days on, or prior to, the index date. For the POWER study, mOCS data were collected via a patient‐reported questionnaire about medication use at the time of baseline questionnaire completion. Patients who indicated a change in medication in a follow‐up visit were asked to indicate what the change was since the last study visit.

^i^ One patient with mOCS dose > 100 mg was excluded from this descriptive summary.

^j^ Available in BE‐REAL (Belgium) and BEEPS (Switzerland) only.

^k^ ACQ score includes both ACQ‐6 scores based on the average over six items (POWER [Canada], BE‐REAL [Belgium], and imPROve [Germany]) and ACQ‐5 scores based on the average over five items (BEEPS [Switzerland]).

The median (interquartile range [IQR]) age at benralizumab treatment initiation was 58.0 years (48.0–66.0); 76.3% of patients had an age at asthma diagnosis of ≥ 18 years, and 49.2% were female. At the index date (Week 0), 35.5% of patients were receiving mOCS. The median daily dose of prednisolone equivalent was 20 mg at index. Baseline disease‐related characteristics were broadly similar between biologic‐naïve and biologic‐experienced patients, although biologic‐experienced patients were slightly younger and more likely to be female (Table 1). Among biologic‐experienced patients (*n* = 89) with drug‐specific data available (*n* = 63), 63.5% (40/63) and 28.6% (18/63) had previously received mepolizumab and omalizumab, respectively. Maintenance asthma treatments at baseline, overall and by previous biologic experience, are listed in Table S1.

Overall, 25.0% (134/535) of patients had a normal BMI, and 68.8% (368/535) had BMI values that corresponded to being overweight or obese (34.4% overweight [*n* = 184], 34.4% obese [*n* = 184]). The proportion of patients in each BMI group was similar, regardless of previous biologic status (Table 1).

Treatment adherence was available in BE‐REAL, imPROve, and BEEPS; of 363 patients with 12 months of follow‐up data, 285 (78.5%) were still receiving benralizumab at 12 months. Benralizumab treatment patterns, adherence, and reasons for discontinuation are reported in Table S2.

### 3.2 Asthma Symptoms

#### 3.2.1 Change From Baseline in ACQ Score Over 56 Weeks

**FIGURE 1:**
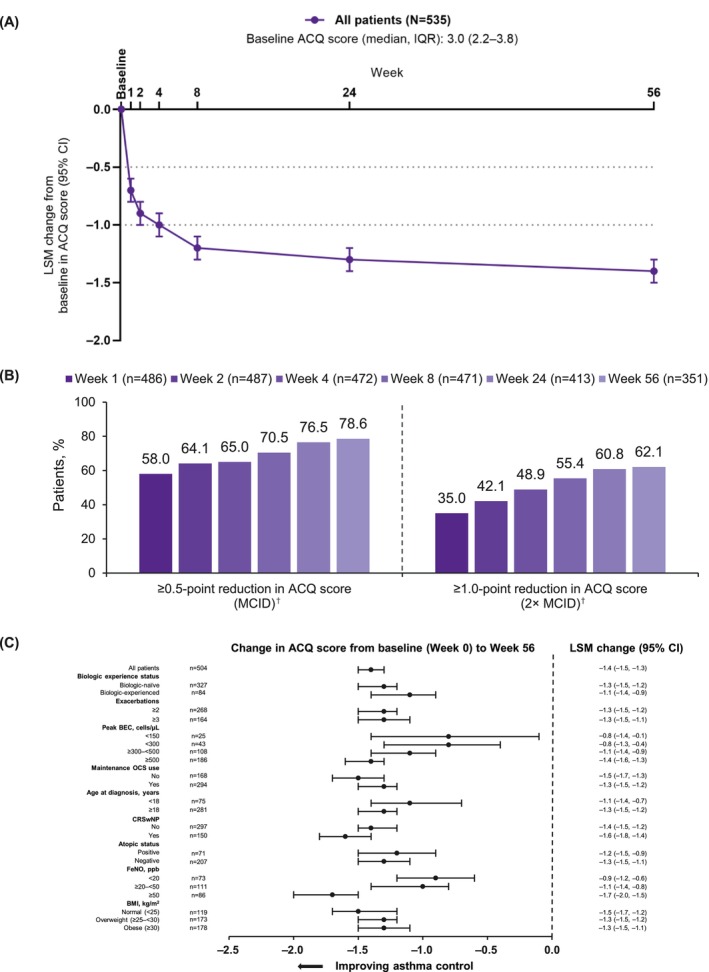
(A) ACQ score change from baseline over 56 weeks for patients overall. (B) ACQ responders^†^ from Week 1 to 56 (vs. baseline [index, Week 0]). (C) ACQ score change from baseline (index, Week 0) over 56 weeks, by key baseline clinical characteristics. Baseline was defined as Week 0 (index, initiation of benralizumab); Week 1 was the first time point at which ACQ scores were recorded, so there may have been an improvement earlier than this time point. Dotted lines represent the MCID and 2× MCID. The ACQ score includes both ACQ‐6 scores, based on an average of six items in Canada, Belgium, and Germany, and ACQ‐5 scores, based on an average of five items in Switzerland. ‘Biologic‐experienced’ status refers to any previous use of biologic treatment before the index date. During the 12‐month baseline period, exacerbations, BEC, and maintenance OCS use were monitored. CRSwNP, atopic status, and BMI were based on medical record data as of the index date. The FeNO value was taken from the most recent measurement within the baseline period, based on medical record data. ^†^MCID refers to the smallest change in ACQ score (≥ 0.5‐point reduction) that would lead to a change in the patient's medical management; 2× MCID refers to a ≥ 1.0‐point reduction in ACQ score. ACQ, Asthma Control Questionnaire; ACQ‐5, five‐item Asthma Control Questionnaire; ACQ‐6, six‐item Asthma Control Questionnaire; BEC, blood eosinophil count; BMI, body mass index; CI, confidence interval; CRSwNP, chronic rhinosinusitis with nasal polyposis; FeNO, fractional exhaled nitric oxide; IQR, interquartile range; LSM, least squares mean; MCID, minimal clinically important difference; OCS, oral corticosteroid.

Overall, the median (IQR) ACQ score at index was 3.0 (2.2–3.8). The greatest improvement in ACQ score occurred between index (Week 0) and Week 1, with further improvement up to Week 56 (Figure 1A). Changes from index to Week 1 and Week 56 in LSM ACQ scores were −0.7 (95% CI –0.8, −0.6, *n* = 504) and −1.4 (95% CI –1.5, −1.3, *n* = 504), respectively. At Week 1, 58.0% (282/486) of patients had clinically meaningful improvements of ≥ 0.5 points (MCID) and 35.0% (170/486) of ≥ 1 point (2× MCID, Figure 1B). At Week 56, 78.6% (276/351) had an improvement of MCID and 62.1% (218/351) of ≥ 2× MCID. Patients had early and sustained improvements regardless of previous biologic use status, but improvements were more pronounced among biologic‐naïve than biologic‐experienced patients (Figure S2A). Patients with well‐controlled asthma at Week 56 showed an improvement in ACQ scores, with the mean (SD) score decreasing from 2.5 (1.2) at baseline to 0.3 (0.3). In contrast, those whose asthma remained not well‐controlled experienced a more modest improvement, with mean (SD) scores decreasing from 3.3 (1.1) at baseline to 2.6 (0.9) at Week 56 (Figure S2B).

#### 3.2.2 Change From Baseline to Week 56 in ACQ Score by Baseline Characteristics

In subgroup analyses, significantly improved asthma control and clinically relevant improvements were seen across all baseline characteristic subgroups (Figure 1C). From baseline to Week 56, changes in LSM ACQ scores were −1.3 (95% CI –1.5, −1.2, *n* = 327) in biologic‐naïve patients and −1.1 (95% CI –1.4, −0.9, *n* = 84) in biologic‐experienced patients. ACQ score improvements were more pronounced in patients with higher peak BEC, no mOCS use at baseline, no atopy, with CRSwNP, higher FeNO, and normal BMI; improvements were greatest in patients with no mOCS use, with CRSwNP, FeNO ≥ 50 ppb, and normal BMI.

#### 3.2.3 Changes in Asthma Symptom Control Status to Week 56

**FIGURE 2:**
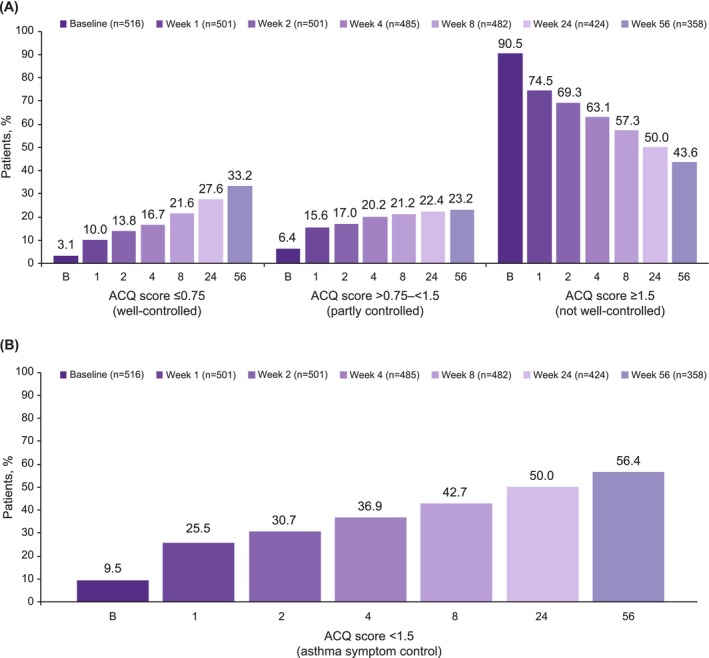
(A) Asthma symptom control status (well‐controlled, partly controlled, not well‐controlled), and (B) proportion of patients with asthma symptom control (ACQ score < 1.5) at baseline (index, Week 0) and at Weeks 1, 2, 4, 8, 24, and 56. Baseline was defined as Week 0 (initiation of benralizumab). ACQ score included both ACQ‐6 scores based on an average of six items (POWER [Canada], BE‐REAL [Belgium], and imPROve [Germany]), and ACQ‐5 scores based on an average of five items (BEEPS [Switzerland]). ACQ, Asthma Control Questionnaire; ACQ‐5, five‐item Asthma Control Questionnaire; ACQ‐6, six‐item Asthma Control Questionnaire.

From index (Week 0) to Week 1, there was a more than threefold increase in the proportion of patients with well‐controlled asthma (Week 0, 3.1%; Week 1, 10.0%), followed by a steady improvement in asthma control up to Week 56 (33.2%, Figure 2A). Similarly, 6.4% had partly controlled asthma at index and 15.6% at Week 1, rising steadily to 23.2% at Week 56. The number of patients with not well‐controlled asthma dropped from 90.5% at index to 74.5% at Week 1, and approximately halved to 43.6% by Week 56.

The proportion of patients with controlled asthma (ACQ score < 1.5) more than doubled from index (9.5%) to Week 1 (25.5%) and steadily increased to 56.4% at Week 56 (Figure 2B).

### 3.3 Other Clinical Outcomes: Annualised Exacerbation Rate, mOCS Use and Lung Function

Relative reduction in AER from baseline (3.0) to Week 56 (0.48) was 84.0% overall (*n* = 393) and 86.7% and 74.4% in biologic‐naïve (*n* = 310) and biologic‐experienced (*n* = 83) patients, respectively (Figure S3).

The median (IQR) and mean percentage changes in daily mOCS dose from index (Week 0) to Week 56 among all patients using mOCS at the index date (*n* = 190, 35.5%) with mOCS daily dosage available at Week 56 (*n* = 172, 32.1%) was −96.7% (−100.0% to 0.0%) and −53.7%, respectively (Figure S4). By Week 56, 50.0% (*n* = 86) had eliminated mOCS use completely (59.7% [71/119] and 31.0% [13/42] in biologic‐naïve and biologic‐experienced patients, respectively).

Lung function data were available in 385/535 patients at baseline (72.0% of all patients, from BE‐REAL, imPROve, and BEEPS). Data on lung function improvements in the overall population and by biologic status are presented in Figure S5. In patients with available data (*n* = 82) at Week 56, a pre‐bronchodilator FEV_1_ improvement of ≥ 100 mL was found in 68.3% of patients, and an improvement of ≥ 200 mL was found in 56.1% of patients.

### 3.4 Clinical Remission

**FIGURE 3:**
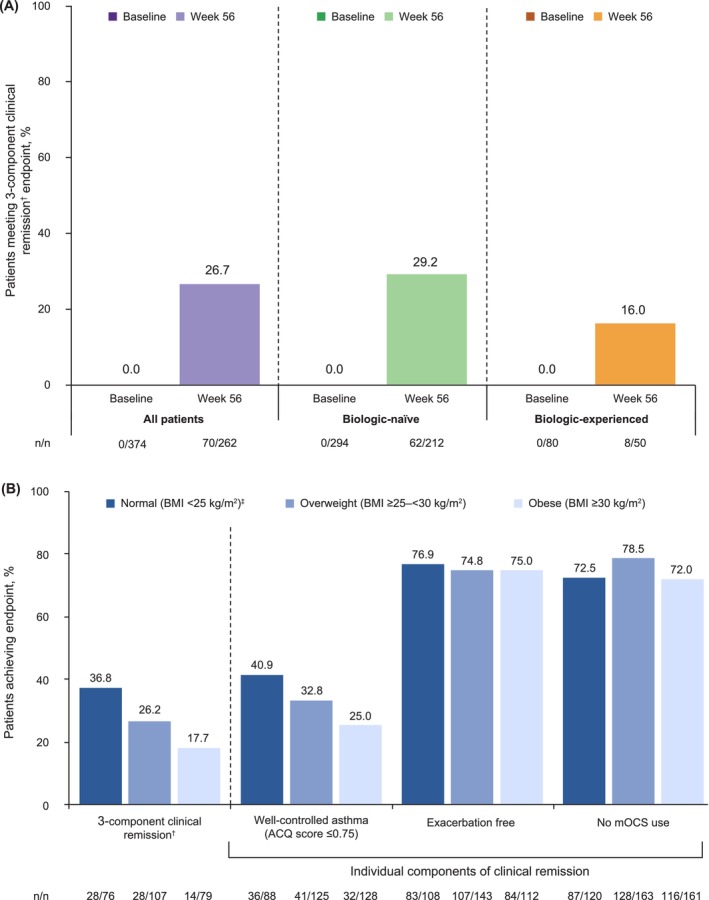
(A) Proportion of patients meeting 3‐component (composite) clinical remission at baseline (Week 0) and Week 56, using ACQ score ≤ 0.75 for the overall population and according to patients' previous biologic experience. (B) Proportion of patients meeting 3‐component (composite) clinical remission using ACQ score ≤ 0.75, and the individual components of remission, at Week 56, according to patients' BMI group. ^†^3‐component (composite) remission was defined as ACQ score ≤ 0.75, no exacerbations, and no mOCS use. Exacerbations were assessed during the 12 months prior to index (baseline period) and from the index date to Week 56 (follow‐up period). Asthma symptom control and mOCS use status were assessed at index (Week 0) and Week 56. ACQ score included both ACQ‐6 scores based on an average of six items (POWER [Canada], BE‐REAL [Belgium], and imPROve [Germany]), and ACQ‐5 scores based on an average of five items (BEEPS [Switzerland]). ^‡^Includes nine patients with BMI < 18.5 kg/m^2^. ACQ, Asthma Control Questionnaire; ACQ‐5, five‐item Asthma Control Questionnaire; ACQ‐6, six‐item Asthma Control Questionnaire; BMI, body mass index; mOCS, maintenance oral corticosteroids.

The criteria for 3‐component clinical remission were met in 0% (0/374) and 26.7% (70/262) of patients at baseline and Week 56, respectively (Figure 3A); at Week 56, 29.2% (62/212) of biologic‐naïve patients and 16.0% (8/50) of biologic‐experienced patients met the criteria. Using the less stringent criterion of ACQ score < 1.5 in the composite definition, 46.2% of biologic‐naïve patients and 26.0% of biologic‐experienced patients met the criteria at Week 56 (Figure S6).

Patients who were overweight or obese were substantially less likely to meet the 3‐component clinical remission endpoint: 26.2% and 17.7%, respectively, versus 36.8% with below‐normal or normal BMI (Figure 3B; Figure S7; see also Figure S8). These differences were largely driven by the considerably smaller proportion of patients with obesity meeting the well‐controlled asthma (ACQ score ≤ 0.75) component at Week 56 (25.0% [32/128]) compared with those with below‐normal or normal BMI (40.9% [36/88]) or who were overweight (32.8% [41/125]).

#### 3.4.1 Individual Components of Remission

**FIGURE 4:**
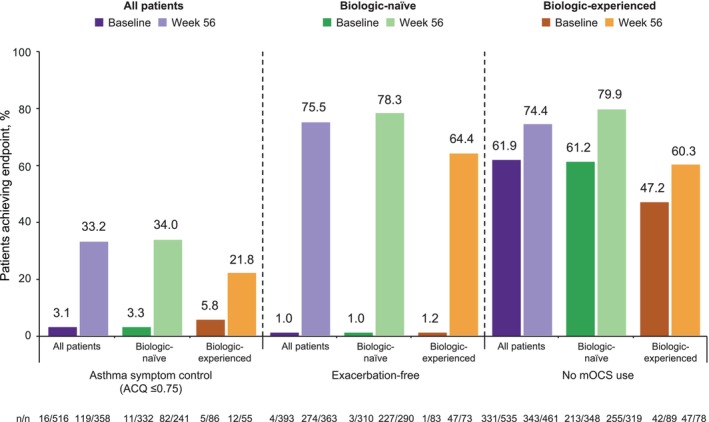
Proportion of patients meeting the individual components of remission of well‐controlled asthma,^†^ No exacerbations^‡^ and no mOCS use^§^ at baseline and Week 56 for the overall population and according to patients' previous biologic experience. ACQ score included both ACQ‐6 scores based on an average of six items (POWER [Canada], BE‐REAL [Belgium] and imPROve [Germany]), and ACQ‐5 scores based on an average of five items (BEEPS [Switzerland]). Exacerbations were assessed during the 12 months prior to index (baseline period) and from the index date to Week 56 (follow‐up period). Asthma symptom control and OCS use status were assessed at baseline (Week 0) and Week 56. ^†^ACQ score ≤ 0.75, measured at index (Week 0) and at Week 56. ^‡^For baseline, exacerbations were measured during the 12‐month baseline period; for Week 56, exacerbations were measured from baseline (Week 0) to Week 56. ^§^At index (Week 0) and at Week 56. ACQ, Asthma Control Questionnaire; ACQ‐5, five‐item Asthma Control Questionnaire; ACQ‐6, six‐item Asthma Control Questionnaire; mOCS, maintenance oral corticosteroids.

Well‐controlled asthma (ACQ score ≤ 0.75) was achieved in 33.2% (119/358) of patients at Week 56 versus 3.1% (16/516) at Week 0. More biologic‐naïve than biologic‐experienced patients achieved well‐controlled asthma at Week 56 (34.0% [82/241] and 21.8% [12/55], respectively). At Week 56, 75.5% (274/363) of patients were exacerbation‐free, compared with 1.0% (4/393) at baseline. The percentage of patients with no mOCS use was 61.9% (331/535) at index (Week 0) and increased to 74.4% (343/461) at Week 56 (Figure 4).

#### 3.4.2 Association Between Baseline Characteristics and Clinical Remission

Results of the univariable analysis of key baseline clinical characteristics and clinical remission at Week 56 are presented in Table S3 and Figure S8. Biologic‐naïve, no mOCS use, better asthma symptom control (ACQ score < 1.5), higher peak BEC, lower immunoglobulin E, presence of CRSwNP, shorter asthma duration, and lower BMI at baseline were positively associated with meeting criteria for clinical remission at Week 56.

Results of the multivariable analysis concerning clinical remission at Week 56 are presented in Figure S9. Lower mOCS dose (OR 0.86 [95% CI 0.77, 0.96]), shorter asthma duration (OR 0.87 [95% CI 0.72, 1.05]), better asthma symptom control (ACQ score < 1.5) (OR 2.05 [95% CI 0.90, 4.64]), and higher peak BEC (OR 1.89 [95% CI 1.38, 2.60]) at baseline were positively associated with meeting the criteria for clinical remission at Week 56.

### 3.5 Improvements From Baseline in Nasal Polyposis VAS Score

Median (IQR) nasal polyposis VAS score for nasal blockage/nasal congestion (*n* = 93) at baseline was 6.4 (4.2–8.5) in the overall population with CRSwNP; baseline VAS scores for difficulty with sleeping (*n* = 88), headache/pressure on face (*n* = 91), and loss of sense of smell (*n* = 89) are also presented in Figure S10. Overall, 50%–61% of the patients had improvements (> 1 unit) from baseline in all symptoms assessed, which were seen as early as Week 24 and steadily increased to between 58% and 77% at Week 56 (Figure S10).

## 4 Discussion

XALOC‐2 is the first prospective real‐world, large‐scale study of benralizumab in patients with SEA, with or without biologic experience. The early and frequent assessment of asthma symptoms over 56 weeks, reported directly by patients at or between visits, allowed the assessment of early changes in asthma symptom control, as well as in 3‐component clinical remission in patients with SEA. These results complement the findings of the retrospective XALOC‐1 program [7], using prospective data collected using validated, standardised questionnaires.

Benralizumab triggers the rapid and near‐complete depletion of blood eosinophils, with a speed of onset very similar to that of oral prednisolone [8]. In the current study, patients receiving benralizumab had clinically meaningful improvements in ACQ symptom control scores as early as Week 1 post initiation (the earliest time point assessed), regardless of previous biologic use, with improvements continuing through Week 56. Additionally, improvements in symptom control were accompanied by an improvement in ratings of bothersome symptoms from nasal polyps in patients with CRSwNP at baseline, an important comorbidity that impacts the achievement of remission [26, 27, 28].

The criteria for 3‐component remission (including a strict criterium for well‐controlled asthma: ACQ score ≤ 0.75) were met in 26.7% of all patients at Week 56. This proportion is in line with remission rates reported in previously published studies of patients with severe asthma treated with different biologics that used ACQ score ≤ 0.75 as the threshold for asthma symptom control [18].

Clinical remission has been assessed in both clinical trials and real‐world studies of patients with severe asthma; however, cross‐study comparisons are constrained by variability in biologic therapies, differing definitions of remission, and heterogeneity in disease severity [5, 25, 29, 30]. Reported remission rates can be strongly influenced by the remission criterium chosen for good asthma symptom control [18]. A recent systematic review and meta‐analysis in patients with severe asthma treated with biologics (encompassing studies with differing criteria for good asthma symptom control) reported pooled clinical remission rates of 38% using a 3‐component definition and 30% with a 4‐component definition [17].

In the ongoing US‐based Phase 4 interventional PASSAGE study evaluating tezepelumab, which incorporated lung function into the remission criteria, a remission rate of 41.7% was observed at 1 year [31]. Similarly, a retrospective real‐world study from the United Kingdom reported a 36% 4‐component remission rate with tezepelumab at 1 year [32]. In the prospective, real‐world REALITI‐A study, approximately one‐third of patients treated with mepolizumab achieved 3‐component remission—defined as the absence of OCS and exacerbations, with well‐controlled symptoms (ACQ score < 1.5)—after 1–2 years of treatment [33]. Long‐term data for dupilumab are also encouraging: in a *post hoc* analysis of the Phase 3 QUEST trial and its open‐label extension study, TRAVERSE, 37.2% of patients receiving dupilumab met a 4‐component clinical remission definition at Week 52, compared with 22.2% in the placebo group [34]. Notably, 42.8% of patients maintained on dupilumab for 2 years achieved remission [34]. Additionally, in a real‐world Japanese cohort study conducted between April 2019 and November 2022, 44.9% of patients receiving dupilumab achieved remission based on symptom control and exacerbation‐free status irrespective of lung function; 26.5% achieved remission (FEV_1_ ≥ 70%) while 24.5% met the more stringent criterion of FEV_1_ ≥ 80% [35].

A recent review of benralizumab, which consolidated *post hoc* remission data from both clinical trials and real‐world studies, demonstrated similar and consistent remission trends, reinforcing the feasibility of achieving clinical remission in patients with SEA treated with benralizumab [36]. Indeed, at the end of the SIROCCO and CALIMA Phase 3 trials (12 months), 37.7% of patients achieved remission based on the 3‐component criteria, and 32.0% based on the 4‐component criteria [36].

Although differences in study design, patient populations, and remission definitions limit direct comparisons, the collective evidence supports clinical remission as an achievable and durable outcome across multiple biologic therapies. Nevertheless, the lack of standardisation in clinical remission definitions continues to pose challenges for cross‐study comparisons.

In addition to demographic and clinical factors associated with achieving clinical remission, a subset of patients fails to attain remission due to a lack of therapeutic benefit or clinically meaningful response to their current biologic treatment [37, 38]. A key contributing factor is the underlying heterogeneity of asthma phenotypes, which influences individual responses to specific biologic therapies [1]. These observations underscore the need for more personalised, phenotype‐driven treatment approaches. Future research should focus on identifying reliable predictors of response across biologic classes to facilitate optimal therapy selection and enhance the likelihood of achieving remission in a broader population of patients with SEA. Indeed, given the heterogeneity in treatment response across different clinical outcomes, a recently published study by Khaleva et al. introduced novel patient‐centred composite outcome scores (CompOsite iNdexes For Response in asthMa [CONFiRM]) for both paediatric and adult patients with severe asthma, integrating clinical parameters and quality‐of‐life measures to offer a validated, multidimensional tool for assessing biologic treatment response [39]. CONFiRM has demonstrated strong external validity and discriminative ability, supporting its use in providing a more holistic understanding of patient response and enabling standardised evaluation of biologics effectiveness across studies [39].

As in XALOC‐1 [7, 22] and *post hoc* analyses of RCTs of benralizumab in patients with severe uncontrolled asthma [25, 28], improvements in asthma symptom control and 3‐component clinical remission in the current study were seen in patients with a range of baseline characteristics important to therapeutic decision‐making in the real world, including in both biologic‐naïve and biologic‐experienced patients. Lower mOCS dose, lower BMI, better asthma symptom control (ACQ score < 1.5) and higher peak BEC at baseline were positively associated with meeting the criteria for 3‐component clinical remission at Week 56, using the less stringent cutoff for asthma symptom control (ACQ score < 1.5) in the remission criteria. Concerning BMI, patients who were overweight or obese were substantially less likely to achieve clinical remission, particularly those with obesity. The primary driver of this was the considerably smaller proportion of patients with obesity meeting the asthma symptom control (ACQ score ≤ 0.75 or < 1.5) component at Week 56, compared with those with normal BMI or who were overweight. This observed relationship between BMI and 3‐component clinical remission is consistent with previous literature, which indicates that higher BMI is typically more strongly linked to poorer scores on patient‐reported outcomes (like the ACQ) than to asthma severity [23] and that the patient‐reported symptom burden may reflect the interaction of comorbidities with asthma, or symptoms of comorbidities, like anxiety and/or depression [40].

Lung function criteria are included in some, but not all, guideline definitions of asthma remission and, when present, they are not always clearly defined [18, 41, 42]. Since this study used data from routine clinical practice, there was limited availability of lung function data. Furthermore, it is likely that the coronavirus disease 2019 (COVID‐19) pandemic further limited the availability of these data. When available, lung function data may also be biased towards patients with more severe disease who may be more likely to have these measured frequently at follow‐up visits. Consequently, lung function was not included in the remission composite in this study owing to limited availability of data. The 3‐component definition of remission used in this study aligns with the wider literature [16], and is endorsed by various national guidelines [16, 18, 41, 42].

Overall, the findings were consistent with those of XALOC‐1 [7]. Remission was more achievable for patients with lower disease burden prior to benralizumab initiation, adding weight to the potential benefits of earlier treatment intervention in these patients, as suggested in the XALOC‐1 analysis. In the current analysis, no patients met the remission criteria at baseline.

This analysis has several key strengths. First, the use of prospectively collected data at early and frequent time points allowed for robust assessment of clinically relevant outcomes, including the validated ACQ. The application of a standardised definition of clinical remission further supports the long‐term effectiveness of benralizumab in improving clinical outcomes and achieving remission in a broad real‐world patient population with SEA. The inclusion of both biologic‐naïve and biologic‐experienced patients reflects real‐world practice, while the large sample size (535 patients) provided sufficient statistical power to detect clinically meaningful differences. Patient enrollment across four countries enhances the generalizability of the findings, with > 86% of patients contributing 12 months of follow‐up data. Notably, data on asthma symptom control were continuously collected during the COVID‐19 pandemic, as ACQ scores were recorded by patients at home. Finally, the use of rigorous statistical methodology, including multivariable analyses to identify predictors of remission, further strengthens the validity of the study design.

However, several limitations of this analysis should be acknowledged. The absence of a control arm limits the ability to draw causal inferences regarding the effectiveness of benralizumab compared with other biologics or standard care. All XALOC‐2 component studies were conducted during the COVID‐19 pandemic, which may have affected visit attendance, data completeness, and certain outcome measures. Variability in data collection methods across the four participating countries introduced heterogeneity and, as is common in studies relying on patient‐reported and electronic health data, some data were not collected using standardised protocols. Missing data were observed for a subset of patients, particularly with respect to the three components of clinical remission, and the availability of data varied across assessment timepoints. Due to limited lung function data, this parameter could not be included in the remission definition. Additionally, there was no formal integrated analysis of safety or adverse events; available data were restricted to individual studies, with limited data collection on healthcare resource utilisation, treatment adherence data, or reasons for discontinuation. Finally, the 56‐week follow‐up period is relatively short in the context of severe asthma. Longer‐term outcomes from the 112‐week integrated analysis will be published separately.

## 5 Conclusions

Clinically meaningful improvements in asthma symptom control began as early as 1 week after benralizumab initiation, regardless of previous biologic use, and further improved over the following year of treatment, with more than a quarter of patients achieving 3‐component clinical remission. Such findings indicate that targeting eosinophilic inflammation in SEA with benralizumab is associated with a rapid and meaningful response in asthma symptom control across patients with key clinically relevant characteristics important to decision‐making in daily practice.

## Author Contributions

All authors were involved in the study implementation, data acquisition, and/or interpretation of the data. All authors critically reviewed the manuscript, approved the final version for submission, and agree to be accountable for all aspects of this work. Erika Penz: concept or design of the study, acquisition of data, data interpretation. Thomas Rothe: concept or design of the study, acquisition of data, data interpretation. Lieven Dupont: acquisition of data, analysis of data, data interpretation. Trung N. Tran: concept or design of the study, data interpretation. Andrew Menzies‐Gow: analysis of data, data interpretation. Anat Shavit: concept or design of the study, acquisition of data, data interpretation. David Cohen: analysis of data, data interpretation. Tanja Plate: concept or design of the study, acquisition of data, data interpretation. Sheena Kayaniyil: analysis of data, data interpretation. An Herreman: concept or design of the study, data interpretation. Claudio Schuoler: concept or design of the study, analysis of data, data interpretation. Benjamin Emmanuel: concept or design of the study, analysis of data, data interpretation. Marek Lommatzsch: concept or design of the study, acquisition of data, data interpretation.

## Conflicts of Interest

E.P. has received grants/research support from AstraZeneca, Sanofi, GSK, CIHR, SHRF, Respiratory Research Centre, Saskatchewan Lung, Saskatchewan Centre for Patient‐Oriented Research, and Saskatchewan Cancer Agency. She has received speakers' bureau/honoraria from AstraZeneca, Boehringer Ingelheim, COVIS Pharma, GSK, Sanofi Genzyme, and the International Centre for Evidence Based Medicine. She has received consulting fees from AstraZeneca and GSK. T.R. has received payments for talks and advisory boards from AstraZeneca, GSK, Novartis, Sanofi, and Teva. In addition, he has received grants/research support from AstraZeneca. L.D. declares no conflicts of interest. T.N.T., A.M.‐G., A.S., D.C., T.P., S.K., A.H., C.S. and B.E. are or were employees of AstraZeneca at the time of this work, and may own stock in AstraZeneca. A.S. is currently an employee of Boehringer Ingelheim. M.L. reports grants for research or clinical trials, paid to his institution, from AstraZeneca, Deutsche Forschungsgemeinschaft (DFG), and GSK; and consulting fees, travel expenses, or honoraria for lectures from ALK, Allergopharma, Apontis, AstraZeneca, Berlin‐Chemie, Boehringer Ingelheim, Chiesi, GSK, HAL Allergy, Leti, Novartis, MSD, Sanofi, Stallergenes, and Teva.

## Supporting information


**Data S1:** cea70162‐sup‐0001‐DataS1.docx.

## Data Availability

Data underlying the findings described in this manuscript may be obtained in accordance with AstraZeneca's data sharing policy described at https://astrazenecagrouptrials.pharmacm.com/ST/Submission/Disclosure.
