# Supplementary material for: Early and Sustained Asthma Control and Remission in Real‐World Patients With Severe Eosinophilic Asthma Treated With Benralizumab: XALOC‐2
Source: Clin Exp Allergy. 2025 Oct 29;56(3):212–26. doi: 10.1111/cea.70162 (PMC13006152; doi:10.1111/cea.70162)
Supplement: Supplementary file 1 — Data S1: cea70162‐sup‐0001‐DataS1.docx. [file CEA-56-212-s001.docx]

# **Supplementary material**

Penz *et al.* ‘Early and Sustained Asthma Control and Remission in Real-World Patients With Severe Eosinophilic Asthma Treated With Benralizumab: XALOC-2’

**Contents**

**[Supporting Information:METHODS S1](#_Ref14550709)** [Per study patient inclusion and exclusion criteria 4](#_Ref14550709)

**[Supporting Information:METHODS S2](#_Ref14874373)** [Definitions, calculations and subgroup analyses used 7](#_Ref14874373)

[**TABLE S1** Maintenance asthma treatments at baseline, for the overall population and according to patients’ previous biologic experience. 11](#_Ref14160018)

[**TABLE S2** Benralizumab treatment patterns, adherence, and reasons for discontinuation.. 12](#_Ref14627724)

[**TABLE S3** Univariable analysis of baseline demographics and clinical characteristics among patients who met 3-component (composite) clinical remission and those who did not at Week 56 14](#_Ref14483025)

[**FIGURE S1** XALOC-2 study design. 17](#_Ref14796257)

[**FIGURE S2** (A) ACQ score change from baseline (index, Week 0) over 56 weeks according to patients’ previous biologic experience and (B) ACQ score change from baseline (index, Week 0) over 56 weeks by responder status. 18](#_Ref14585006)

[**FIGURE S3** Relative reduction in AER from baseline to Week 56 (A) for the overall population and according to patients’ previous biologic experience and (B) by key baseline clinical characteristics.. 19](#_Ref14627813)

[**FIGURE S4** (A) Median and (B) mean percentage change in mOCS dose from baseline (index date, Week 0) to Week 56 among patients with mOCS use at baseline, and (C) median and (D) mean mOCS daily dose at baseline (index date, Week 0) and Week 56 for the overall population and according to patients’ previous biologic experience. 20](#_Ref14346347)

[**FIGURE S5** Proportion of patients with improvement from index in pre-BD FEV_1_ of
(A) ≥100 mL and (B) ≥200 mL at Week 24 and Week 56, for the overall population and according to patients’ previous biologic experience.. 21](#_Ref14430289)

[**FIGURE S6** Proportion of patients meeting (A) 3-component (composite) clinical remission, and the individual components of remission of (B) asthma symptom control, (C) no exacerbations, and (D) no mOCS use, using ACQ score <1.5 (sensitivity analysis) at baseline (Week 0) and Week 56, for the overall population and according to patients’ previous biologic experience. 22](#_Ref14243039)

[**FIGURE S7** Proportion of patients meeting 3-component (composite) clinical remission endpoint using ACQ score ≤0.75 at Week 56, and the individual components of remission, according to patients’ BMI group: (A) biologic-naïve and (B) biologic-experienced. 24](#_Ref14290791)

[**FIGURE S8** Univariable analysis of baseline demographics and clinical characteristics among patients who met 3-component (composite) clinical remission and those who did not at Week 56. 26](#_Ref14572149)

[**FIGURE S9** 3-component (composite) clinical remission multivariable logistic regression analysis at Week 56. Asthma symptom control was defined as ACQ score <1.5. Demographics and characteristics were recorded at baseline. 3-component clinical remission: ACQ score <1.5, no exacerbations and no mOCS use. 2](#_Ref14531914)8

[**FIGURE S10** Proportion of patients with improvement from baseline in nasal polyposis VAS scores. 29](#_Ref14465513)

[**Study contributors** 30](#_Ref14448044)

## **Supporting Information: METHODS S1** Per study patient inclusion and exclusion criteria

| **Study (country) and recruitment period**^†^ | **Inclusion criteria** | **Exclusion criteria** |
| --- | --- | --- |
| **POWER  (Canada)**  November 2018–April 2022 | - ≥18 years of age - Benralizumab naïve - Uncontrolled SEA, meeting one of the following:   - BEC ≥300 cells/µL AND ≥2 clinically significant exacerbations in the past 12 months   - BEC ≥150 cells/µL AND treated chronically with OCS   - Sputum eosinophil levels ≥3%   - Previous biologic therapy for SEA AND met the above BEC prior to initiation of the previous biologic - ACQ-6 score ≥1.5 | - Current enrolment in any interventional clinical study other than a national asthma registry - Previous treatment with benralizumab - Concomitant non-SEA lung disease (including asthma-COPD overlap) |
| **BE-REAL  (Belgium)**  December 2019–February 2022 | - ≥18 years of age - Benralizumab naïve   - ≥ 30 days from last dose of previous biologic - Physician-confirmed diagnosis of severe uncontrolled asthma, meeting one of the following:   - ≥2 hospitalizations or ER treatments for severe asthma in the last 12 months   - ≥2 asthma exacerbations in the last 12 months (asthma worsening requiring ≥3 days SCS and/or an ER visit or hospitalization) - High-dose ICS + LABA maintenance treatment - FEV_1_ <80%, demonstrated by spirometry in the past 12 months - Peripheral BEC ≥300 cells/µL in the last 12 months AND at benralizumab initiation | - Current enrollment in any interventional clinical study except for patients who are in parallel documented in the Belgian Severe Asthma Registry - Previous treatment with benralizumab - Any concurrent biologic asthma therapy excluding stable allergen immunotherapy - Concomitant non-SEA lung disease |
| **imPROve  (Germany)**  December 2019–December 2023 | - ≥18 years of age - Physician-confirmed diagnosis of severe uncontrolled asthma according to ATS/ERS^1^ and local German guidelines - Benralizumab naïve   - ≥ 30 days from last dose of previous biologic | - Participation in an observational trial that may influence assessments for the current study OR participation in an RCT in the last three months - Previous treatment with benralizumab - Any concurrent biologic asthma therapy excluding stable allergen immunotherapy - Concomitant non-SEA lung disease - Pregnancy or lactation |
| **BEEPS  (Switzerland)**  June 2019–January 2023 | - ≥18 years of age - Physician-confirmed diagnosis of severe uncontrolled asthma according to ATS/ERS guidelines^1^ - High-dose ICS + LABA maintenance treatment - ≥2 exacerbations in the last 12 months - Peripheral BEC ≥300 cells/µL | - Current enrollment in any interventional clinical study other than a national asthma registry - Concomitant non-SEA lung disease |

^†^First Subject In to Last Subject In. Study duration (First Subject In to Last Subject Last Visit): POWER: November 2018– May 2024; BE-REAL: December 2019–May 2024; imPROve: December 2019–December 2023; BEEPS: December 2023– January 2023.

ACQ-6, six-item asthma control questionnaire; ATS, American Thoracic Society; BEC, blood eosinophil count; COPD, chronic obstructive pulmonary disease; ER, emergency room;
ERS, European Respiratory Society; FEV_1_, forced expiratory volume in one second;
ICS, inhaled corticosteroids; LABA, long-acting β_2_ agonist; OCS, oral corticosteroids;
RCT, randomized controlled trial; SCS, systemic corticosteroids; SEA, severe eosinophilic asthma.

## **Supporting Information: METHODS S2** Definitions, calculations and subgroup analyses used

| **Definitions of clinical outcomes**   - Previous biologic use   - Biologic-naïve: No previous biologic treatment for severe asthma recorded during the baseline period   - Biologic-experienced: Patients who received ≥1 biologic treatment for asthma during the 12-month baseline period - Asthma symptom control^†^   - Well-controlled: ACQ score ≤0.75   - Partly controlled: ACQ score >0.75–<1.5   - Not well-controlled: ACQ score ≥1.5   - Controlled asthma: ACQ score <1.5 - MCID in ACQ score   - A reduction from baseline (at the index date [Week 0] or the most recent measurement in the 12-month baseline period) to Week 56 matching or exceeding –0.5 units in the ACQ score - Asthma exacerbation   - A worsening of asthma leading to one of the following: (1) Use of SCS for ≥3 days or a temporary increase in a stable background dosage of oral corticosteroids,  (2) emergency department or urgent care visit (<24 hours) due to asthma that required SCS or (3) inpatient admission to hospital (≥24 hours) due to asthma - Pre- and post-BD FEV_1_:^‡^   - Measurement at the index date or the most recent measurement in the 12-month baseline period, and measurement at Week 56 - mOCS dose   - Calculated as the mean daily dosage over the past 30 days on, or prior to, the target date of the specified visit (index date [Week 0], Week 56) |
| --- |
| **Calculations used for statistical analyses**   - AER   - Total number of severe asthma exacerbations occurring in the sample/total duration of follow-up [days]) × 365.25   - The 12-month exacerbation analysis contains patients who discontinued before Week 56 or completed the Week 24 follow-up visit - Relative reduction in AER   - Baseline AER (calculated for the 12-month baseline period): Follow-up AER (calculated for the period from the index date to Week 56)/baseline AER × 100 - Percentage change in mOCS dose at Week 56 from the index date (Week 0) (patient-level)   - mOCS dose at Week 56 (calculated as the mean daily dosage over the past 30 days on or prior to the target date of the specified visit, Week 56): mOCS dose at baseline]/mOCS dose at baseline × 100 |
| **Asthma exacerbations were evaluated for the following baseline subgroups**   - Biologic-experience status in the 12-month baseline period (biologic-naïve and biologic-experienced [including previous experience with omalizumab, mepolizumab, dupilumab and reslizumab]) - mOCS use during the 12-month baseline period (yes, no) - Peak BEC in the 12-month baseline period (<150, <300, ≥300–<500, ≥500 cells/µL) - Number of exacerbations in the 12-month baseline period (≥2, ≥3) - Age at asthma diagnosis (<18, ≥18 years) - Most recent FeNO measurement in the 12-month baseline period (<20, ≥20, 20–<50, <50, ≥50 ppb) - Atopic status (positive, negative; based on medical records available at the index date) - Presence of concomitant CRSwNP (yes, no; based on medical records available at the index date) - BMI (<25 kg/m^2^ [normal], ≥25–<30 kg/m^2^ [overweight] and ≥30 kg/m^2^ [obese]) |

Baseline was defined as the 12-month period before the index date (Week 0; first administration of benralizumab) for exacerbations, and as index date (Week 0) for no maintenance oral corticosteroid (mOCS) use and asthma symptom control. The follow-up period was the period from the index date to Week 56. The target visits specified for the current analyses are the index date and Week 56. Data for ACQ, asthma exacerbations, mOCS and FEV_1_ were also collected at Week 24; in BEEPS (Switzerland), these were collected at Week 16, but they are included with Week 24 data for readability. ^†^ACQ-6^2^ was used in POWER (Canada), BE-REAL (Belgium) and imPROve (Germany); ACQ-5^2^ was used in BEEPS (Switzerland). For POWER, ACQ-6 data were captured via telephone for Weeks 1 and 2 (only). Patients could opt to complete all ACQ-6 questionnaires electronically, except for the baseline visit (Week 0). For BE-REAL, ACQ-6 questionnaires could be completed on paper either during the visit (except for Weeks 1 and 2), at home or at the general practitioner. For imPROve, ACQ-6 was captured using an ePRO device for Weeks 1 and 2; however, if a physician and patient agreed to self-administration at home utilizing the Fasenra® pen or pre-filled syringe, patients were able to complete all the ACQ-6 questionnaires at home. For BEEPS, ACQ-5 questionnaires could be completed either during the visit or at home. ^‡^Lung function measurements were captured only if they were part of the routine clinical practice.
ACQ, Asthma Control Questionnaire; ACQ-5, five-item Asthma Control Questionnaire;
ACQ-6, six-item Asthma Control Questionnaire; AER, annualized exacerbation rate;
BD, bronchodilator; BEC, blood eosinophil count; BMI, body mass index; CRSwNP, chronic rhinosinusitis with nasal polyposis; ePRO, electronic patient-reported outcomes; FeNO, fractional exhaled nitric oxide; FEV_1_, forced expiratory volume in one second; MCID, minimal clinically important difference; mOCS, maintenance oral corticosteroids; SCS, systemic corticosteroids.

## **TABLE S1** Maintenance asthma treatments at baseline, for the overall population and according to patients’ previous biologic experience.^†^

| **Patients, n (%)** | **All patients (*n*=393)** | **Biologic-naïve (*n*=310)** | **Biologic-experienced (*n*=83)** |
| --- | --- | --- | --- |
| ICS | 371 (94.4) | 290 (93.5) | 81 (97.6) |
| LABA | 353 (89.8) | 273 (88.1) | 80 (96.4) |
| LAMA | 196 (49.9) | 156 (50.3) | 40 (48.2) |
| ICS/LABA | 345 (87.8) | 266 (85.8) | 79 (95.2) |
| LTRA^‡^ | (n=149) 55 (36.9) | (n=109) 41 (37.6) | (n=40) 14 (35.0) |

^†^Available in BE-REAL (Belgium), imPROve (Germany) and BEEPS (Switzerland). ^‡^Available in BE-REAL (Belgium) and BEEPS (Switzerland). ICS is calculated as the average daily dose over the past 30 days on, or prior to, the target date of the specified visit.
ICS, inhaled corticosteroid; LABA, long-acting β_2_ agonist; LAMA, long-acting muscarinic antagonist; LTRA, leukotriene receptor antagonist.

## **TABLE S2** Benralizumab treatment patterns, adherence, and reasons for discontinuation.^†^

| **Variables** | **All patients  (*n*=393)** | **Biologic-naïve  (*n*=310)** | **Biologic-experienced  (*n*=83)** |
| --- | --- | --- | --- |
| Patients with 12 months of follow-up, *n* (%) | 363 (92.4) | 290 (93.5) | 73 (88.0) |
| Patients still on benralizumab at 12 months, *n* (%)^‡^ | 285 (78.5) | 227 (78.3) | 58 (79.5) |
| Benralizumab treatment duration |  |  |  |
| Median (IQR), days | 408.0 (297.0–674.0) | 413.5 (302.0–677.0) | 395.0 (247.0–582.0) |
| <3 months, *n* (%)^§^ | 36 (33.3) | 24 (28.9) | 12 (48.0) |
| ≥3–≤6 months, *n* (%)^§^ | 32 (29.6) | 26 (31.3) | 6 (24.0) |
| ≥7–≤12 months, *n* (%)^§^ | 40 (37.0) | 33 (39.8) | 7 (28.0) |
| Adherence to benralizumab, *n* (%) |  |  |  |
| 100% of expected injections | 320 (81.4) | 252 (81.3) | 68 (81.9) |
| ≥75% of expected injections | 344 (87.5) | 272 (87.7) | 72 (86.7) |
| ≥60% of expected injections | 385 (98.0) | 306 (98.7) | 79 (95.2) |
| ≥50% of expected injections | 392 (99.7) | 310 (100) | 82 (98.8) |
| <50% of expected injections | 1 (0.3) | 0 (0) | 1 (1.2) |
| Reasons for discontinuation at 12 months, *n* (%)^¶^ |  |  |  |
| Withdrawal by subject or of consent | (n=97); 15 (15.5) | (n=74); 10 (13.5) | (n=23); 5 (21.7) |
| Loss to follow-up | (n=97); 12 (12.4) | (n=74); 8 (10.8) | (n=23); 4 (17.4) |
| Lack of efficacy | (n=97); 11 (11.3) | (n=74); 8 (10.8) | (n=23); 3 (13.0) |
| Adverse event | (n=97); 3 (3.1) | (n=74); 1 (1.4) | (n=23); 2 (8.7) |
| Other | (n=97); 56 (57.7) | (n=74); 47 (63.5) | (n=23); 9 (39.1) |

^†^Available in BE-REAL (Belgium), imPROve (Germany) and BEEPS (Switzerland). ^‡^Percentage of patients with 12 months’ follow-up. ^§^Percentages are based on the number of patients not on benralizumab at 12 months (n=108), which includes patients who discontinued prior to 12 months and patients whose treatment did not continue to 12 months. ^¶^Assessed at 12 months; percentage of patients who had discontinued at 12 months.

IQR, interquartile range.

## **TABLE S3** Univariable analysis of baseline demographics and clinical characteristics among patients who met 3-component (composite) clinical remission and those who did not at Week 56

| **Variable** | **Clinical remission  (*n*=111)** | **Non-clinical remission  (*n*=151)** | ***p*-value** |
| --- | --- | --- | --- |
| Female, *n* (%) | 63 /111 (56.8) | 76/151 (50.3) | 0.303 |
| Age at start of benralizumab treatment, years, mean (SD)^†^ | 56.1 (14.2) | 55.1 (14.3) | 0.593 |
| Asthma duration, years;  mean (SD)^‡^ | (*n*=108);  15.0 (14.5) | (*n*=147);  17.8 (16.1) | 0.157 |
| BMI, kg/m^2^, mean (SD)^§^ | 27.1 (5.3) | 28.4 (5.4) | 0.053 |
| BMI group, n/N (%) |  |  |  |
| Normal (<25 kg/mg^2^)^¶^ | 39/111 (35.1) | 37/151 (24.5) |  |
| Overweight (≥25–<30 kg/mg^2^) | 48/111 (43.2) | 59/151 (39.1) | 0.479 |
| Obese (≥30 kg/mg^2^) | 24/111 (21.6) | 55/151 (36.4) | 0.009 |
| Smoking history, n/N (%) |  |  |  |
| Ever smoker | 38/111 (34.2) | 58/151 (38.4) | 0.488 |
| Never smoker^¶^ | 73/111 (65.8) | 93/151 (61.6) |  |
| Peak BEC; cells/μL;  mean (SD)^#^ | (*n*=102);  870.5 (705.4) | (*n*=133);  591.5 (517.0) | <0.001 |
| Baseline IgE, IU/mL;  mean (SD)^#^ | (*n*=94);  378.9 (714.0) | (*n*=119);  528.7 (894.0) | 0.191 |
| FeNO, ppb, mean (SD)^\|\|^ | (*n*=82);  53.7 (37.2) | (*n*=105);  46.5 (36.3) | 0.186 |
| Positive atopic status, n/N (%) | 17/87 (19.5) | 27/108 (25.0) | 0.366 |
| Presence of CRSwNP, n/N (%) | 50/97 (51.5) | 43/125 (34.4) | 0.011 |
| Osteoporosis, n/N (%) | 3/28 (10.7) | 12/43 (27.9) | 0.094 |
| Obstructive sleep apnoea, n/N (%) | 9/54 (16.7) | 12/61 (19.7%) | 0.677 |
| Anxiety or depression, n/N (%) | 9/54 (16.7) | 16/62 (25.8%) | 0.235 |
| Any OCS-related comorbidity^††^, n/N (%) | 31/66 (47.0) | 45/78 (57.7) | 0.200 |
| Biologic-naïve status, yes, n/N (%) | 98/111 (88.3) | 114/151 (75.5) | 0.011 |
| Exacerbations during the 12-month baseline period;  mean (SD)^‡‡^ | (*n*=111);  2.7 (2.9) | (*n*=149);  3.1 (3.4) | 0.391 |
| mOCS dose during the 12-month baseline period, mg/day;  mean (SD)^§§^ | (*n*=111);  7.1 (13.4) | (*n*=150);  9.2 (14.5) | 0.238 |
| mOCS use at the index date, mg/day, mean (SD) | 32/110 (29.1) | 78/148 (52.7) | <0.001 |
| Pre-BD FEV_1_, L;  mean (SD)^¶¶^ | (*n*=108);  2.1 (0.8) | (*n*=149);  2.0 (0.8) | 0.261 |
| Pre-BD FEV_1_, % predicted;  mean (SD)^§^ | (*n*=82);  65.8 (19.7) | (*n*=110);  63.5 (47.7) | 0.684 |
| Controlled asthma (ACQ score <1.5), n/N (%) | 24/107 (22.4) | 17/148 (11.5) | 0.021 |
| Participating country, n (%) |  |  |  |
| Switzerland | 23/111 (20.7) | 15/151 (9.9) | 0.021 |
| Germany | 61/111 (55.0) | 97/151 (64.2) | 0.062 |
| Belgium^¶^ | 27/111 (24.3) | 39/151 (25.8) |  |

Clinical remission was defined as patients with asthma symptom control (ACQ score <1.5), no exacerbations and no mOCS at Week 56. Patients from POWER (Canada) were not included as exacerbation results were not available. ^†^Based on five-year increment. ^‡^Based on 10-year increment. ^§^Based on five-unit increment. ^¶^Reference group. ^#^Based on
log_2_-transformed data. ^||^Based on 25-unit increment. ^††^Any OCS-related comorbidities include depression/anxiety, cataracts, glaucoma, obstructive sleep apnoea, type 2 diabetes, cardiovascular disease, or osteoporosis, osteopenia or history of fractures. ^‡‡^Based on one-unit increment. ^§§^Based on 5 mg/daily increment. ^¶¶^Based on 0.5-unit increment.
ACQ, Asthma Control Questionnaire; BD, bronchodilator; BEC, blood eosinophil count; BMI, body mass index; CRSwNP, chronic rhinosinusitis with nasal polyposis; FeNO, fractional exhaled nitric oxide; FEV_1_, forced expiratory volume in 1 second; IgE, immunoglobulin E; mOCS, maintenance oral corticosteroids; OCS, oral corticosteroids; SD, standard deviation.


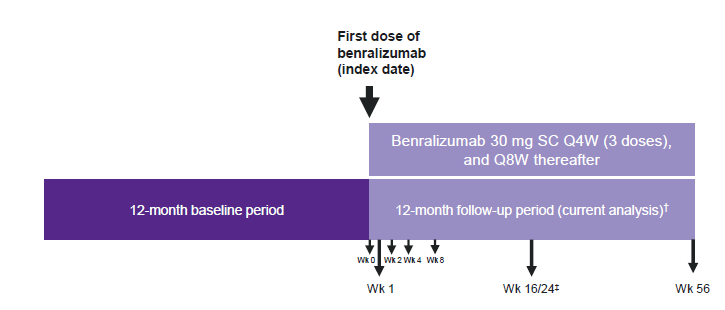


## **FIGURE S1** XALOC-2 study design.

^†^Current analysis; patients in XALOC-2 will be followed up for up to 24 months. ^‡^In the Swiss cohort (BEEPS), patients were assessed at Week 16 instead of Week 24.
Q4W, every 4 weeks; Q8W, every 8 weeks SC, subcutaneous; Wk, week.


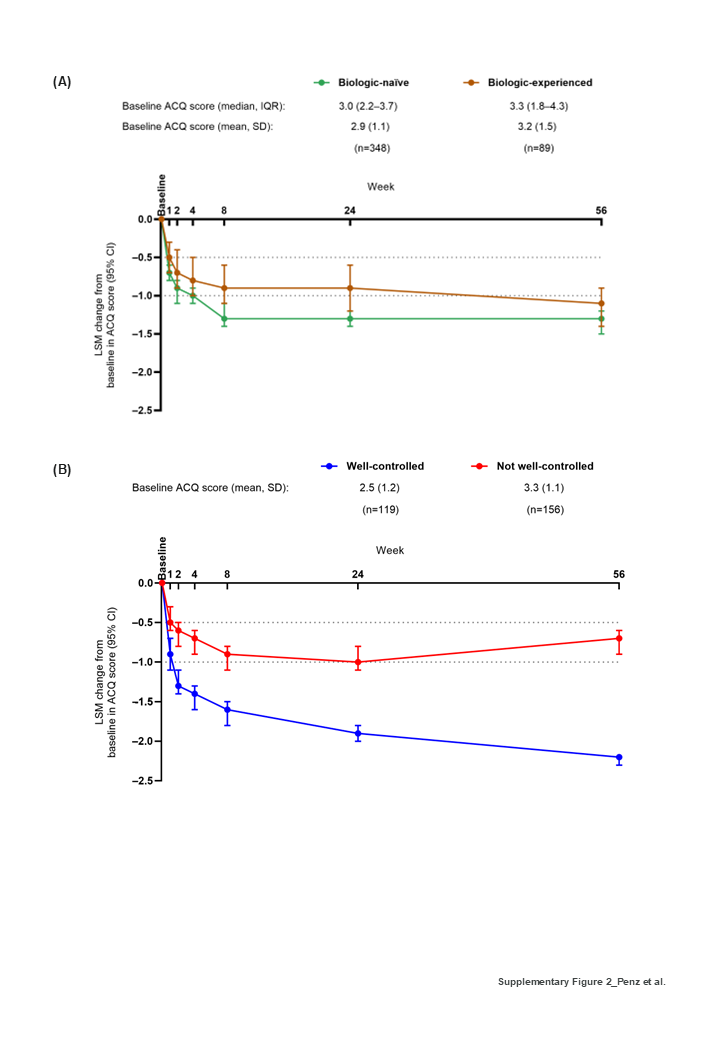


## **FIGURE S2** (A) ACQ score change from baseline (index, Week 0) over 56 weeks according to patients’ previous biologic experience and (B) ACQ score change from baseline (index, Week 0) over 56 weeks by responder status.^†^

^†^At Week 56; well-controlled: ACQ score ≤0.75; not well-controlled: ACQ score ≥1.5.
ACQ, Asthma Control Questionnaire; CI, confidence interval; IQR, interquartile range; LSM, least squares mean; SD, standard deviation.

**
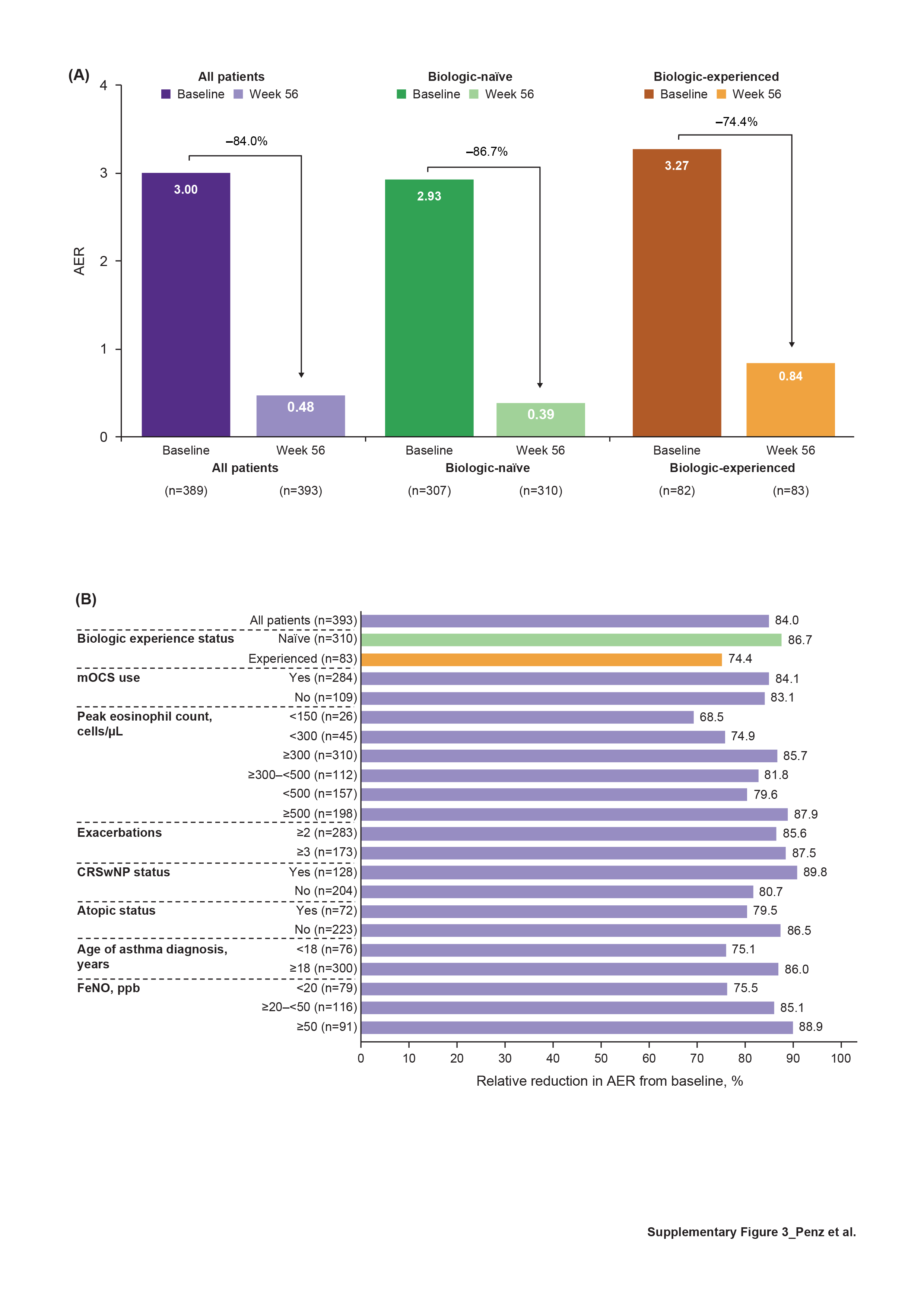
**

## **FIGURE S3** Relative reduction in AER from baseline to Week 56 (A) for the overall population and according to patients’ previous biologic experience and (B) by key baseline clinical characteristics. The 12-month exacerbation analysis contains patients who discontinued before Week 56 or completed the Week 24 follow-up visit. Key baseline clinical characteristics during the 12-month baseline period: mOCS use, BEC, exacerbation, most recent FeNO, age at asthma diagnosis, atopic status and presence of CRSwNP. AER, annualized exacerbation rate; BEC, blood eosinophil count; CRSwNP, chronic rhinosinusitis with nasal polyposis; FeNO, fractional exhaled nitric oxide; mOCS, maintenance OCS; OCS, oral corticosteroids.

**
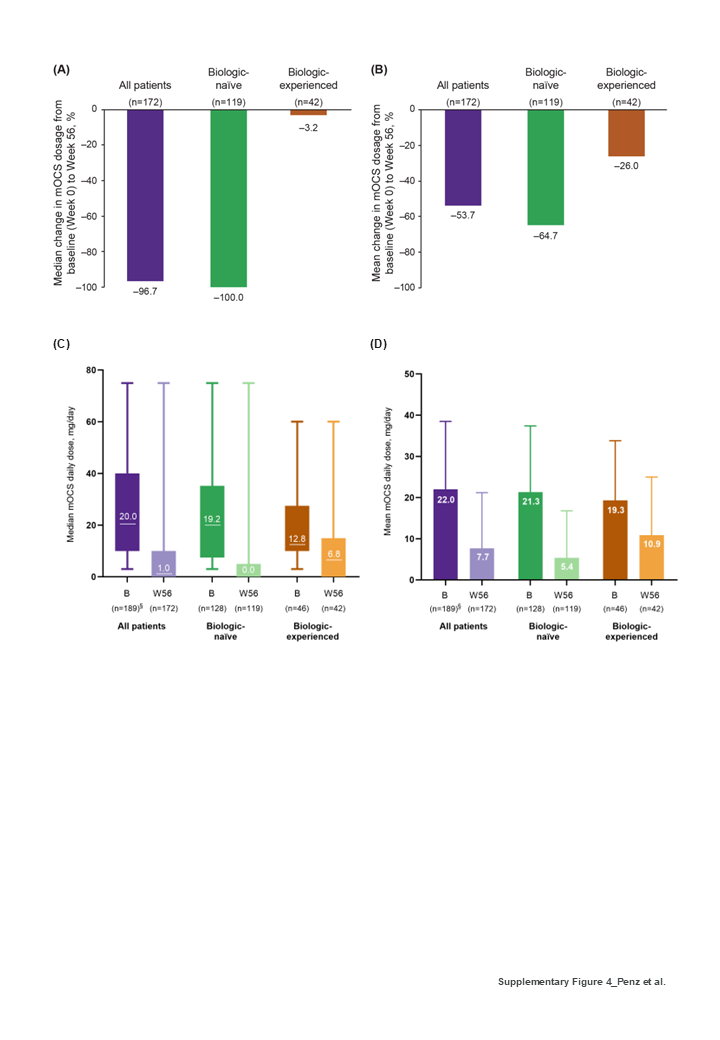
**

## **FIGURE S4** (A) Median and (B) mean percentage change in mOCS dose^†^ from baseline (index date, Week 0) to Week 56 among patients with mOCS use at baseline, and (C) median and (D) mean mOCS daily dose^†^ at baseline (index date, Week 0) and Week 56 for the overall population and according to patients’ previous biologic experience.^‡^

^†^mOCS dose was calculated as a patient’s median daily dose over the past 30 days on, or prior to, the target date of the specified visit; n is the number of patients with mOCS use at the index date (Week 0; n=190, 35.5%) and with follow-up data at Week 56 (n=172, 32.1%). ^‡^There were 98 patients from POWER (Canada) with biologic status missing. ^§^One patient with mOCS dose >100 mg was excluded from the descriptive summary.
mOCS, maintenance oral corticosteroids.


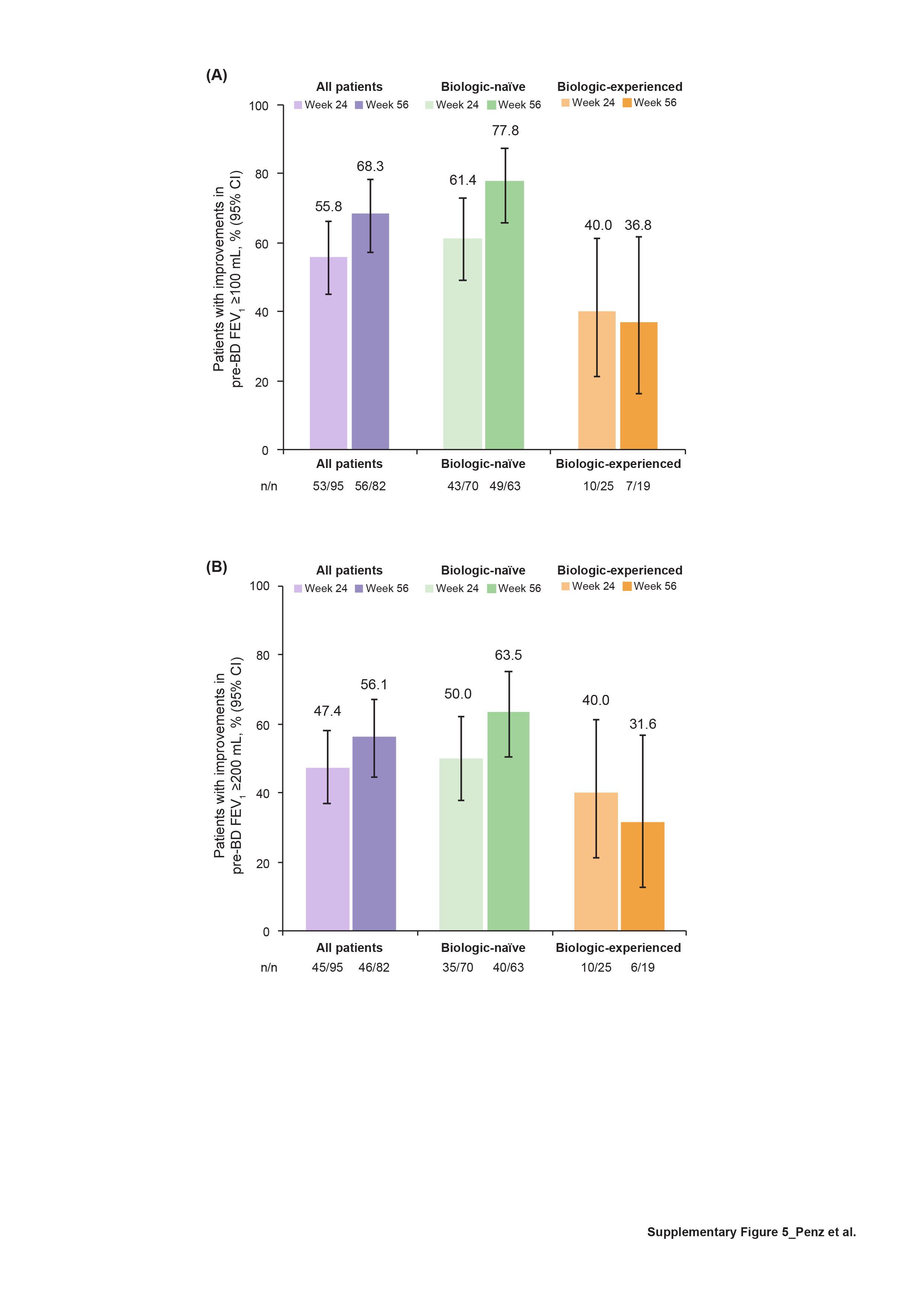


## **FIGURE S5** Proportion of patients with improvement from index in pre-BD FEV_1_ of (A) ≥100 mL and (B) ≥200 mL at Week 24 and Week 56,^†^ for the overall population and according to patients’ previous biologic experience. At Week 56, median (IQR) pre-BD FEV_1_ was 2.3 (1.8–3.0) for all patients (*n*=84/149), 2.4 (1.9–3.0) in biologic-naive (*n*=64/109) and 1.9 (1.4–2.9) in biologic-experienced (*n*=20/40) patients.^†^Week 24 and Week 56 lung function data were available in BE-REAL (Belgium) and BEEPS (Switzerland) only, and they were captured only if part of the routine clinical practice. In BEEPS (Switzerland), these were collected at Week 16, but they are included with Week 24 data for readability. BD, bronchodilator; CI, confidence interval; FEV_1_, forced expiratory volume in one second; IQR, interquartile range.


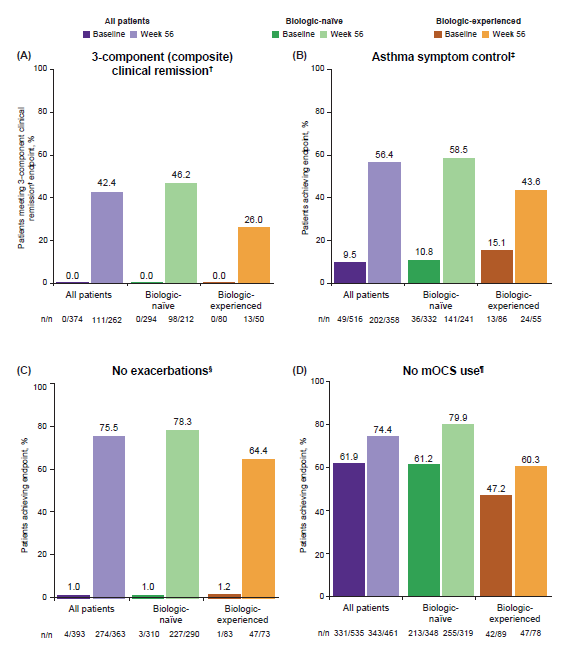


## **FIGURE S6** Proportion of patients meeting (A) 3-component (composite) clinical remission^†^, and the individual components of remission of (B) asthma symptom control^‡^, (C) no exacerbations^§^, and (D) no mOCS use^¶^, using ACQ score <1.5 (sensitivity analysis) at baseline (Week 0) and Week 56, for the overall population and according to patients’ previous biologic experience.

^†^3-component clinical remission: ACQ score <1.5, no exacerbations, and no mOCS use. ^‡^ACQ score <1.5, measured at index (Week 0) and at Week 56. ^§^For baseline, exacerbations were measured during the 12-month baseline period; for Week 56, exacerbations were measured from baseline (Week 0) to Week 56. ^¶^At index (Week 0) and at Week 56. ACQ score included both ACQ-6 scores based on an average of six items (POWER [Canada], BE-REAL [Belgium] and imPROve [Germany]), and ACQ-5 scores based on an average of five items (BEEPS [Switzerland]).
ACQ, Asthma Control Questionnaire; ACQ-5, five-item Asthma Control Questionnaire; ACQ-6, six-item Asthma Control Questionnaire; mOCS, maintenance oral corticosteroids.

^
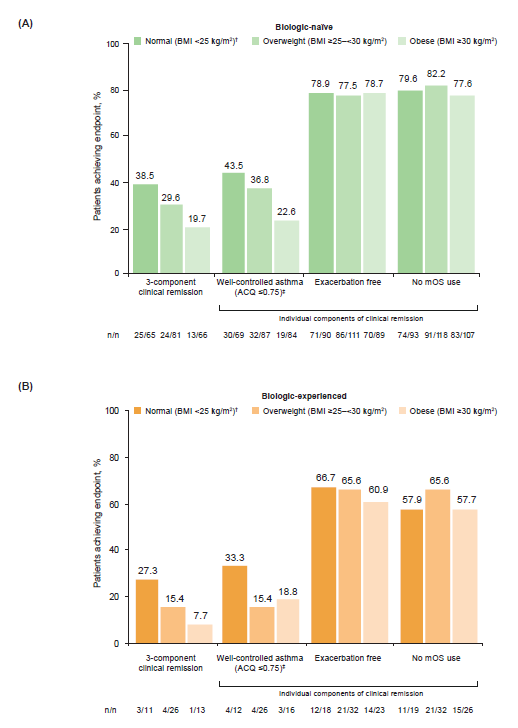
^

## **FIGURE S7** Proportion of patients meeting 3-component (composite) clinical remission endpoint using ACQ score ≤0.75 at Week 56, and the individual components of remission, according to patients’ BMI group: (A) biologic-naïve and (B) biologic-experienced. 3-component clinical remission: ACQ score ≤0.75, no exacerbations, and no mOCS use. Exacerbations were assessed from the index date to Week 56. Asthma symptom control and mOCS use status were assessed at index (Week 0) and Week 56. ^†^Includes nine patients with BMI <18.5 kg/m^2^. ^‡^ACQ score included ACQ-6 scores based on an average over six items (Canada, Belgium, and Germany) and ACQ-5 scores based on average over five items (Switzerland). ACQ, Asthma Control Questionnaire; ACQ-5, five-item Asthma Control Questionnaire; ACQ-6, six-item Asthma Control Questionnaire; BMI, body mass index; mOCS, maintenance oral corticosteroids.

**
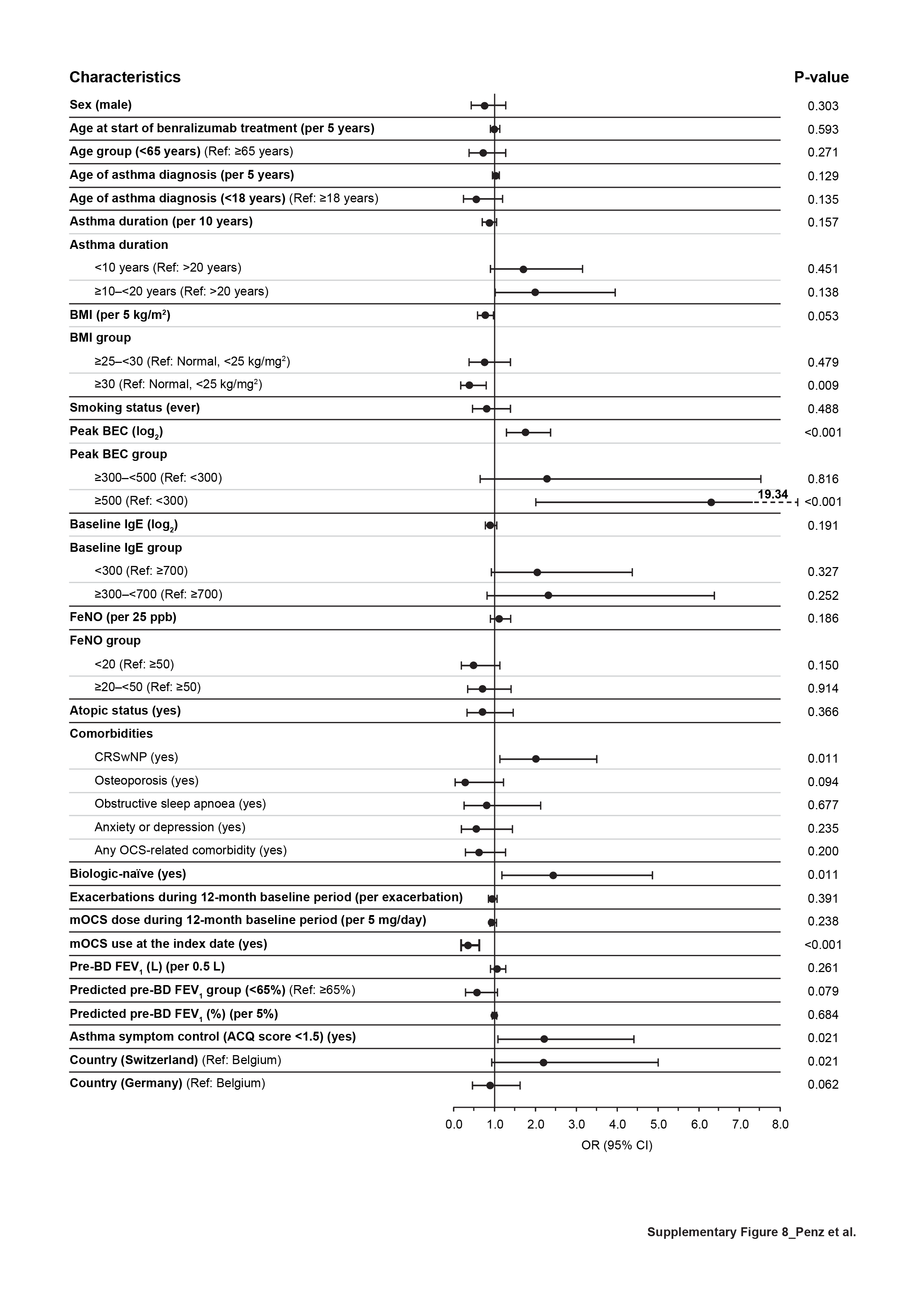
**

## **FIGURE S8** Univariable analysis of baseline demographics and clinical characteristics among patients who met 3-component (composite) clinical remission and those who did not at Week 56.

Asthma symptom control was defined as ACQ score <1.5. Demographics and characteristics were recorded at baseline. 3-component clinical remission: ACQ score <1.5, no exacerbations and no mOCS use.
ACQ, Asthma Control Questionnaire; BD, bronchodilator; BEC, blood eosinophil count; BMI, body mass index; CI, confidence interval; CRSwNP, chronic rhinosinusitis with nasal polyposis; FeNO, fractional exhaled nitric oxide; FEV1, forced expiratory volume in one second; IgE, immunoglobin; mOCS, maintenance OCS; OCS, oral corticosteroids; OR, odds ratio.


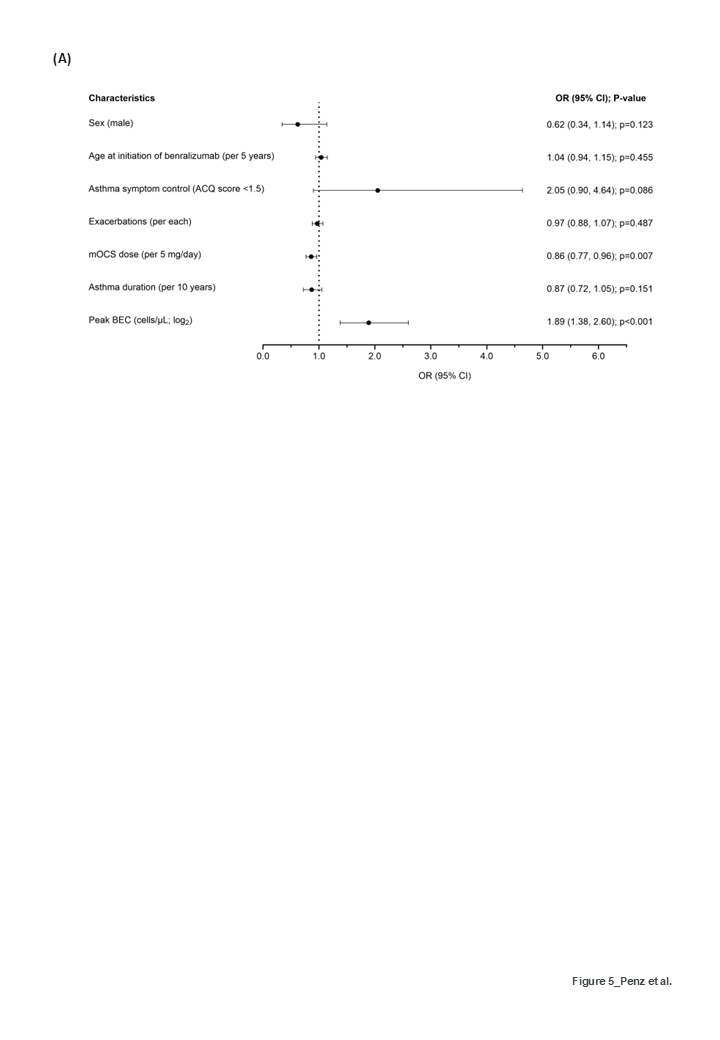


## **FIGURE S9** 3-component (composite) clinical remission multivariable logistic regression analysis at Week 56. Asthma symptom control was defined as ACQ score <1.5. Demographics and characteristics were recorded at baseline. 3-component clinical remission: ACQ score <1.5, no exacerbations and no mOCS use. ACQ, Asthma Control Questionnaire; BEC, blood eosinophil count; CI, confidence interval; mOCS, maintenance oral corticosteroids; OR, odds ratio.


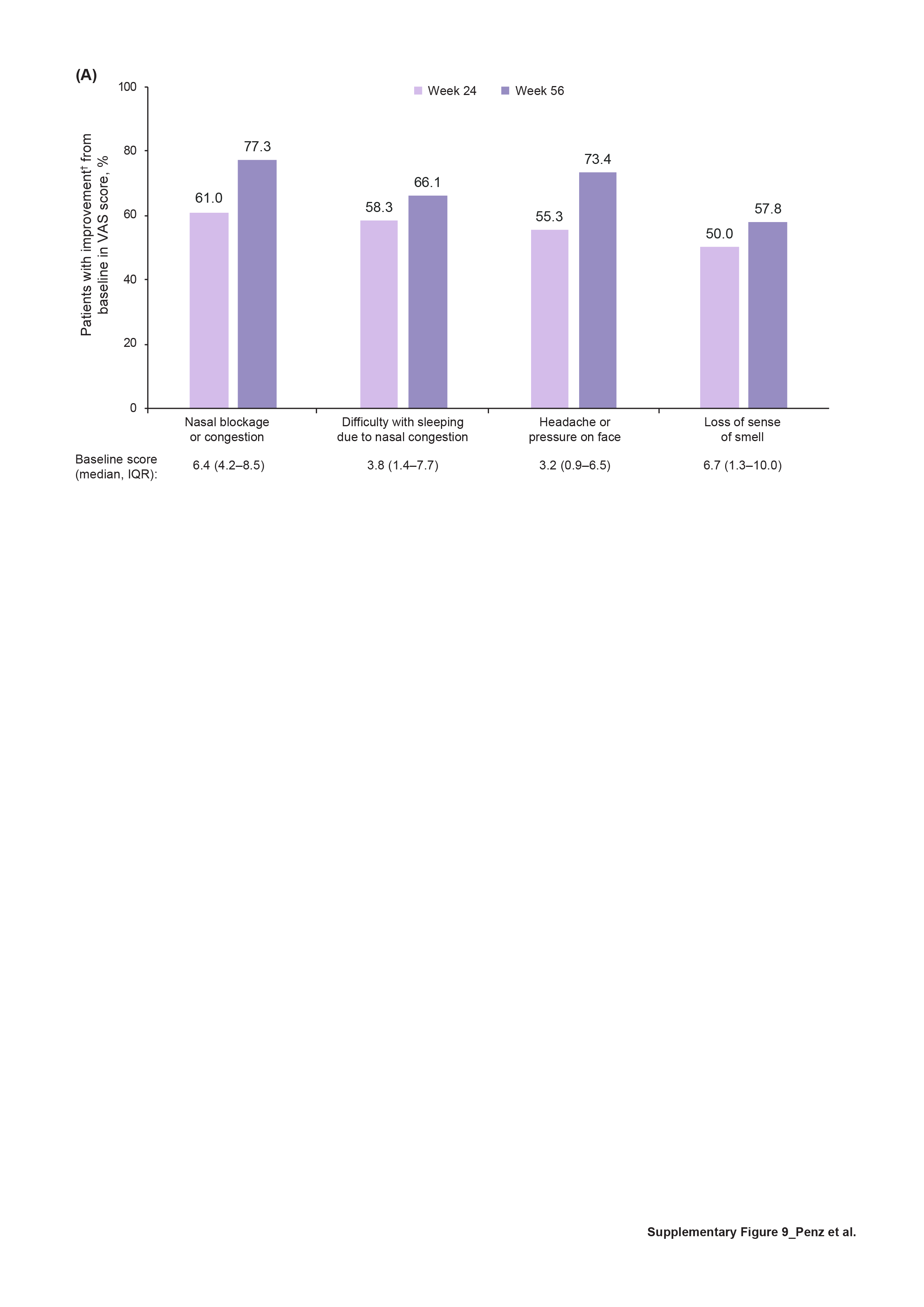


## **FIGURE S10** Proportion of patients with improvement^†^ from baseline in nasal polyposis VAS scores. ^†^Improvement was defined as a change of <0 (i.e. at least one unit; changes were assessed at Week 24 and Week 56 in patients with comorbid nasal polyps at baseline only). Week 24 and Week 56 nasal polyposis VAS scores were available for BE-REAL (Belgium) and imPROve (Germany). The VAS measured how bothersome the symptoms were on a scale of 1–10 (where one is not all bothersome, and 10 is extremely bothersome) over the previous month. IQR, interquartile range; VAS, Visual Analogue Scale.

## **Study contributors**

| **Name^*^** | **Affiliation** | **Country (study)** |
| --- | --- | --- |
| Lancaster, Brett | Department of Medical Affairs, AstraZeneca Canada, Mississauga, Ontario | Canada  (POWER) |
| Mbuagbaw, Lawrence | Biostatistics Unit/The Research Institute, St Joseph's Healthcare—Hamilton, Hamilton, Ontario |  |
| Noorduyn, Stephen G. | GSK, Mississauga, Ontario (formerly of AstraZeneca, Mississauga, Ontario) |  |
| Deschampheleire, Maud | Department of Respiratory Medicine, CHR Citadelle, Liege | Belgium  (BE-REAL) |
| Eger, Katrien | Antwerp University Hospital, Edegem |  |
| Leys, Mathias | Department of Respiratory Medicine, AZ Groeninge, Kortrijk |  |
| Lins, Muriel | AZ Sint-Maarten, Mechelen, Vlaanderen |  |
| Louis, Renaud | CHU de Liege University Hospital Centre Ville, Liege |  |
| Martinot, Jean-Benoit | Institute of Experimental and Clinical Research, UCL Bruxelles Woluwe, Brussels |  |
| Peché, Rudi | Department of Pneumology, Centre Hospitalier Universitaire de Charleroi, Charleroi |  |
| Pillette, Charles | Pneumology Department, Cliniques Universitaires Saint‐Luc, Université catholique de Louvain, Brussels |  |
| Quataert, Charlotte | Real World Evidence, AstraZeneca, Dilbeek |  |
| Dirlam, Dana | IQVIA, Berlin | Germany  (imPROve) |
| Grund, Thorsten | AstraZeneca, Wedel |  |
| Korn, Stephanie | Clinical Research Center, Respiratory Medicine, Institut für Klinische Forschung (IKF) Pneumologie Mainz, Mainz |  |
| Saathoff, Matthias | Alcedis GmbH, Gießen, Hessen |  |
| Watz, Henrik | Velocity Clinical Research Germany GmbH, Berlin |  |
| Bridevaux, Pierre-Olivier | Centre Hospitalier du Valais Romand, Service de Pneumologie, Sion, Switzerland | Switzerland  (BEEPS) |
| Charbonnier, Florian | University Hospital Geneva (HUG), Pulmonology Division, Geneva, Switzerland |  |
| Fiechter, René | Pulmonary Specialty Center, Aathal, Switzerland |  |
| Hauser, Thomas | Immunologie-Zentrum Zürich [Immunology Centre Zurich], Zurich, Switzerland |  |
| Jandus, Peter | University Hospital Geneva (HUG), Immunology and Allergology Division, Geneva, Switzerland |  |
| Leuppi, Jörg D. | Cantonal Hospital Baselland, University Centre of Internal Medicine, Liestal, Switzerland |  |
| Pavlov, Nikolay | Department for Pulmonary Medicine, Allergology and Clinical Immunology, University Hospital Bern, Bern, Switzerland |  |
| Scherer, Thomas | Pulmonary Clinic Bethanien, Zurich, Switzerland |  |
| Stolz, Daiana | University Hospital Basel, Pulmonology Clinic, Basel, Switzerland |  |

^*^Surname, first name

**References**

1 Holguin F, Cardet JC, Chung KF, *et al*. Management of severe asthma: A European Respiratory Society/American Thoracic Society guideline. *Eur Respir J* 2020;55(1):1900588.

2 Juniper EF, O'Byrne PM, Guyatt GH, *et al*. Development and validation of a questionnaire to measure asthma control. *Eur Respir J* 1999;14(4):902-907.
